# RANKL immunisation inhibits prostate cancer metastasis by modulating EMT through a RANKL-dependent pathway

**DOI:** 10.1038/s41598-021-91721-2

**Published:** 2021-06-09

**Authors:** Mineon Park, Yong Jin Cho, Bora Kim, Young Jong Ko, Yuria Jang, Yeon Hee Moon, Hoon Hyun, Wonbong Lim

**Affiliations:** 1 Laboratory of Orthopaedic Research, Chosun University Hospital, Dong-gu, Gwangju, 61452 Republic of Korea; 2 Department of Orthopaedic Surgery, Chosun University Hospital, Dong-gu, Gwangju, 61452 Republic of Korea; 3 Department of Dental Hygiene, Chodang University, Muan County, Jeollanam-do 58530 Republic of Korea; 4 Department of Biomedical Sciences, Chonnam National University Medical School, Gwangju, 61469 Republic of Korea; 5 Department of Premedical Science, College of Medicine, Chosun University, Dong-gu, Gwangju, 61452 Republic of Korea

**Keywords:** Cancer, Molecular biology

## Abstract

Prostate cancer (PCa) morbidity in the majority of patients is due to metastatic events, which are a clinical obstacle. Therefore, a better understanding of the mechanism underlying metastasis is imperative if we are to develop novel therapeutic strategies. Receptor activator of nuclear factor kappa-B (NF-κB) ligand (RANKL) regulates bone remodelling. Thus, agents that suppress RANKL signalling may be useful pharmacological treatments. Here, we used preclinical experimental models to investigate whether an inactive form of RANKL affects bone metastasis in RANKL-induced PCa. RANKL was associated with epithelial–mesenchymal transition (EMT) and expression of metastasis-related genes in PC3 cells. Therefore, we proposed a strategy to induce anti-cytokine antibodies using mutant RANKL as an immunogen. RANKL promoted migration and invasion of PC3 cells through EMT, and induced a significant increase in binding of β-catenin to TCF-4, an EMT-induced transcription factor in PCa cells, via mitogen-activated protein kinase and β-catenin/TCF-4 signalling. Thus, RANKL increased EMT and the metastatic properties of PC3 cells, suggesting a role as a therapeutic target to prevent PCa metastasis. Treatment with mutant RANKL reduced EMT and metastasis of PC3 PCa cells in an experimental metastasis model. Thus, mutant RANKL could serve as a potential vaccine to prevent and treat metastatic PCa.

## Introduction

Prostate cancer (PCa) is the second most common cancer in males and the fifth leading cause of death worldwide^1^. Metastasis of PCa cells to the skeleton occurs in a predictable manner, with lesions tending to appear first in the axial skeleton, followed by appendicular tissues^2^. Considering the effects of PCa on both haematopoiesis and bone structure, bone metastasis is a major cause of morbidity in patients with advanced disease. Replacement of haematopoietic tissue by metastatic PCa cells is associated with anaemia and increased morbidity (mortality). The 5-year survival of most patients with PCa is almost 100%; however, that of PCa patients with metastasis to distant sites is as low as 28%. Thus, PCa is one of the deadliest cancers^3^. Experimental and clinical observations reveal that treatment with anti-resorptive agents suppresses or even prevents PCa metastasis^4–7^, and that accelerated bone turnover stimulates progression of skeletal secondary tumours^8–10^.

During bone metastasis of PCa, cancer cell-derived cytokines stimulate expression of receptor activator of nuclear factor kappa-B ligand (RANKL), which in turn activates bone resorption. RANKL, also known as tumour necrosis factor-related activation-induced cytokine (TRANCE)^11^, osteoprotegerin ligand (OPGL)^12,13^ and osteoclastic differentiation factor (ODF)^14^, interacts with RANK and is involved in all the steps related to tumour development, from initial tumour formation to migration of cancer cells and subsequent metastasis^15^. RANKL is expressed in several tissues, including brain, skin, intestine, skeletal muscle, kidney, liver, lung and mammary tissue; however, expression is very high in bone^16^, lymphoid organs and the vascular system^17^. RANKL binds to RANK on the surface of pre-osteoclasts, activating them and inducing formation of osteoclasts^18^.

Recent studies report expression of RANK and RANKL by various solid tumours, including breast cancer. RANKL accelerates migration and metastasis of cancer cells expressing RANK^19,20^. Furthermore, it protects breast cancer cells from apoptosis in response to DNA damage and controls self-renewal and anchorage-independent growth of tumour-initiating cells^21^. However, it is unclear how RANKL signalling triggers metastasis of PCa. Epithelial-to-mesenchymal transition (EMT), a rapid and often reversible phenotypic change in epithelial cells, is an important phenomenon underlying cancer metastasis. Originally, EMT was described in the context of developmental processes such as heart morphogenesis and mesoderm and neural crest formation. Epithelial cells lose structures involved in cell–cell adhesion (e.g., adherens junctions and desmosomes), modulate their polarity and rearrange their cytoskeleton, which is consistent with the typical switch of intermediate filaments from cytokeratins to vimentin^22^. A recent report shows that the oncogenic c-MYC, Wnt signalling and β-catenin pathways activate the Snail/glycogen synthase kinase-3 (GSK-3) axis and induce EMT^23^.

The concept of exploiting the host’s immune system to treat cancer relies on the ability of immune cells to eliminate malignant cells at the early transformation stage in a process called immune surveillance^24^. Passive anti-cytokine immunotherapy with specific high-affinity antibodies has been tested in animal models and clinical trials of rheumatoid arthritis, multiple sclerosis, inflammatory bowel disease, asthma, Crohn’s disease, psoriasis and other articular autoimmune disorders. This strategy facilitates production of anti-auto-cytokine antibodies by the immune system in response to active vaccination. However, development of a desired antibody response to self-proteins necessitates suppression of immune resistance. Common anti-cytokine vaccines are prepared from autologous proteins that are converted to derivatives that lack biological activity following treatment with glutaraldehyde or formaldehyde^25^. A previous study developed a mouse RANKL mutant (mRANKL-MT) protein and confirmed its ability to inhibit osteoporosis.

Here, we used mRANKL-MT as an immunogen for RANKL-targeting immunotherapy of bone disease and investigated its potential as a cancer vaccine in a mouse model of metastatic cancer. We confirmed the activity of mRANKL-MT and clarified its effects on RANK/RANKL signalling-mediated EMT in transient RANKL-overexpressing cell lines and animal models. We found that mRANKL-MT suppressed RANKL-dependent β-catenin signalling. We also found that mRANKL-MT immunotherapy altered the characteristics of cancer cells and effectively suppressed RANKL-dependent cancer metastasis.

## Results

### Effects of human RANKL (hRANKL) on EMT and metastasis of PC3 cells

To explore the relationship between RANKL and EMT of PC3 cells, we performed cell migration and invasion assays with PC3 cells treated with hRANKL.

**Figure 1:**
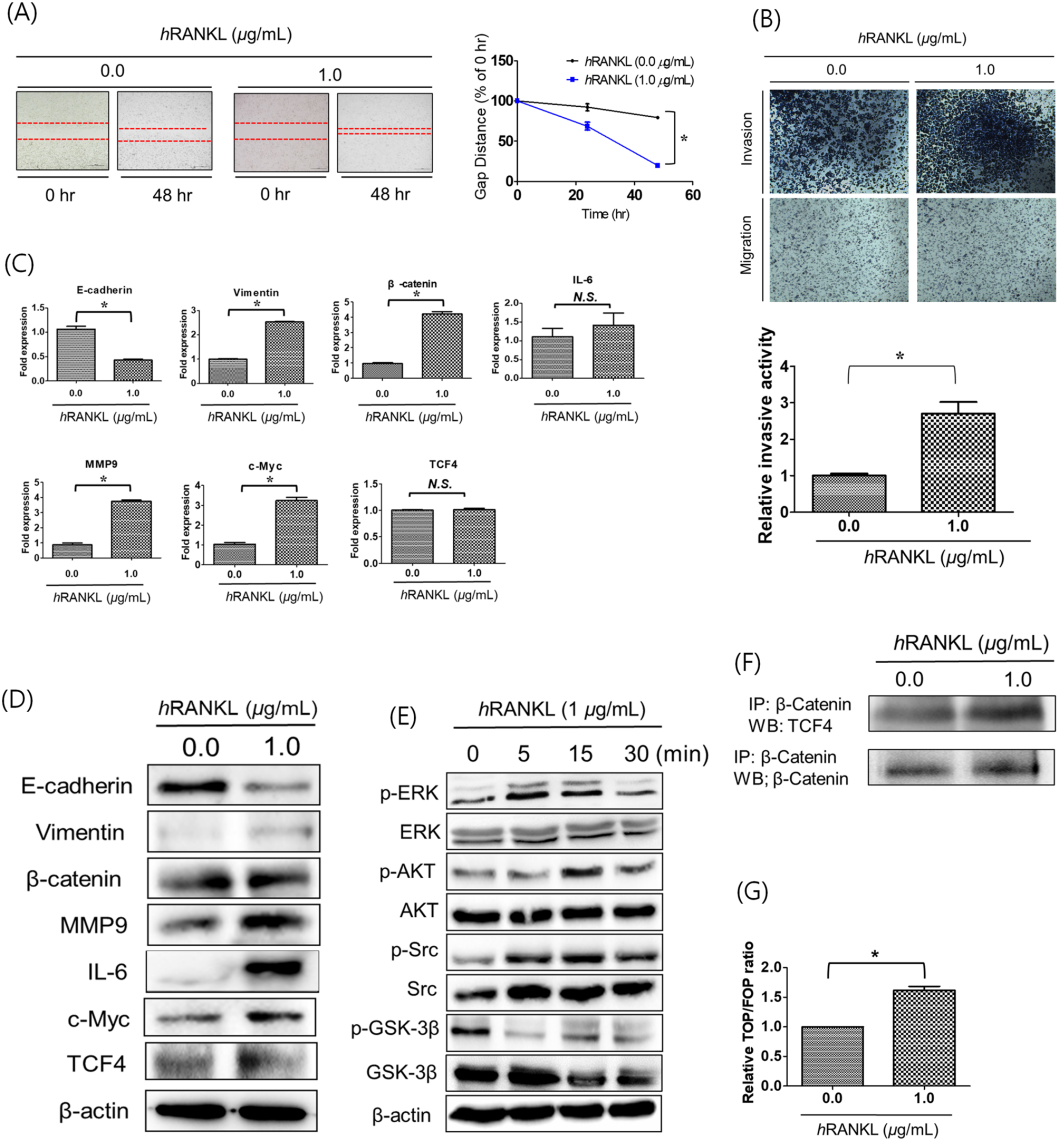
EMT and metastatic properties of PC3 cells following hRANKL treatment. (**A**) Treatment with hRANKL led to a significant increase in the migratory capacity of PC3 cells, as evident from the decrease in the wound gap distance at 48 h. Magnification, × 100; scale bar, 100 μm. The data in the associated graphs are expressed as the mean ± SD. (**B**) Transwell invasion assays were performed to compare the invasiveness of hRANKL-treated PC3 cells and untreated cells. Treatment with hRANKL led to a significant reduction in the invasiveness of PC3 cells. Magnification, × 100; scale bar, 100 μm. Data in the associated graphs are expressed as the mean ± SD. (**C**) Real-time quantitative polymerase chain reaction for the analysis of EMT and metastasis markers in hRANKL-treated PC3 cells. β-Actin was used as a loading control. Significant differences were observed at *p < 0.05, compared with the control. (**D**) Expression of EMT and metastasis-related proteins E-cadherin, Vimentin, β-catenin, MMP-9, IL-6, c-MYC, TCF-4 and β-actin in hRANKL-treated PC3 cells was measured by western blotting. β-actin was used as a loading control. (**E**) Western blot analysis of MAPK phosphorylation in PC3 cells incubated with 1 μg/mL hRANKL for 0, 5, 15 and 30 min. (**F**) Co-immunoprecipitation of β-catenin and TCF-4 in hRANKL-treated PC3 cells. Results are representative of three separate experiments with comparable results. (**G**) TOP/FOP luciferase reporter assays in PC3^Wild^ and PC3^RANKL+^ cells. Significant differences were observed at *p < 0.05, compared with the control.

In the migration assay, wound healing in hRANKL-treated PC3 cells was significantly better than that in control cells (Fig. 1A). Cell invasion also increased significantly following hRANKL treatment (Fig. 1B), although cell proliferations were not increased or decreased (Supple 1).

EMT is closely related to tumour metastasis and progression. Therefore, to determine changes at the molecular level, we measured EMT markers in PC3 cells at the mRNA (Fig. 1C) and protein (Fig. 1D) levels following treatment with hRANKL. Expression of the EMT marker E-cadherin in hRANKL-treated PC3 cells fell significantly, but that of vimentin and β-catenin increased. Expression of MMP-9 and IL-6 (markers of metastasis) were higher in hRANKL-treated cells than in control cells. Expression of E-cadherin protein was significantly lower in hRANKL-treated PC3 cells than in control cells. By contrast, expression of vimentin and β-catenin proteins was significantly upregulated following treatment with hRANKL.

Next, we examined phosphorylation of extracellular signal-regulated kinase (ERK), protein kinase B (AKT), SRC and GSK-3B to investigate the effects of RANKL on mitogen-activated protein kinase (MAPK) and Wnt signalling in PC3 cells (Fig. 1E). PC3 cells treated with hRANKL showed a significant and time-dependent reduction in the level of phosphorylated GSK-3B. A time-dependent increase in SRC and AKT phosphorylation levels was also observed.

Next, we performed co-immunoprecipitation of β-catenin/TCF-4 and TOP/FOP reporter assay to investigate the status of TCF-associated signalling in hRANKL-treated PC3 cells. The results showed a slight increase in TCF-4 levels (Fig. 1F) and TOP/FOP reporter luciferase activity (Fig. 1G) in hRANKL-treated PC3 cells.

Thus, RANKL treatment may trigger metastasis of PC3 cells by suppressing GSK-3B phosphorylation and facilitating Wnt/β-catenin pathway, resulted in EMT progression.

### Overexpression of RANKL modulates EMT and metastasis of PC3 cells

To investigate whether RANKL overexpression stimulates PC3 cell growth in vitro, cells were transiently transfected with an overexpression plasmid containing RANKL. GFP expression by PC3^RANKL^ cells was monitored by fluorescence microscopy (Supp. 2A). We found a significant increase in expression of RANKL mRNA and protein (Supple 2B and 2C).

**Figure 2:**
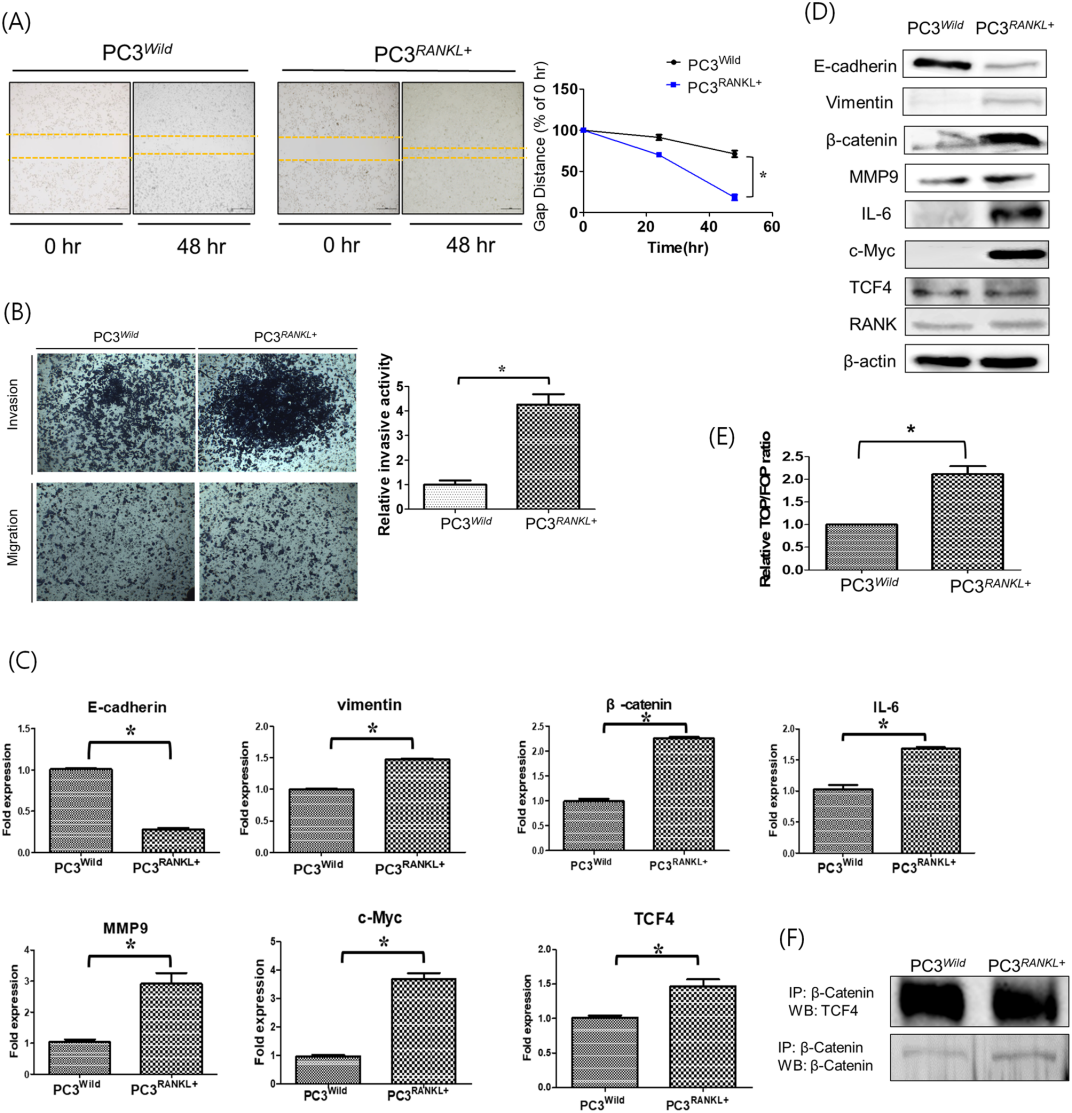
Modulation of the EMT and metastatic properties of PC3 cells following overexpression of RANKL. (**A**) A cell migration assay was performed to compare wound healing in PC3^Wild^ and PC3^RANKL+^ cells. PC3^RANKL+^ cells showed a significant increase in migratory capacity, as evident from the decreased wound gap at 48 h. Magnification, × 100; scale bar, 100 μm. The data in the associated graphs are expressed as the mean ± SD. (**B**) Transwell invasion assays were performed to compare the invasiveness of PC3^Wild^ and PC3^RANKL+^ cells. PC3^RANKL+^ cells showed a significant increase in invasiveness. Magnification, × 100; scale bar, 100 μm. Data in the associated graphs are expressed as the mean ± SD. (**C**) Real-time quantitative polymerase chain reaction for the analysis of EMT and metastasis markers in PC3^RANKL+^ cells. β-Actin was used as a loading control. Significant differences were observed at *p < 0.05, compared with the control. (**D**) Expression of E-cadherin, Vimentin, β-catenin, MMP-9, IL-6 and β-actin in PC3^Wild^ and PC3^RANKL+^ cells, as measured by western blotting. β-Actin was used as a loading control. (**E**) Immunoprecipitation for β-catenin and TCF-4 in PC3^Wild^ and PC3^RANKL+^ cells. Each blot was obtained under the same experimental conditions and the data are representative of three separate experiments with comparable results. (**F**) TOP/FOP luciferase reporter assays in PC3^Wild^ and PC3^RANKL+^ cells. Significant differences were observed at *p < 0.05, compared with the control.

Next, we performed migration and invasion assays using PC3^RANKL^ cells to evaluate the effects of RANKL overexpression on EMT and metastasis. Wound healing was significantly better in PC3^RANKL^ cells than in control cells (Fig. 2A). Also, PC3^RANKL^ cells were significantly more invasive than control cells (Fig. 2B). Analysis of mRNA encoding EMT- and metastasis-related factors in PC3^RANKL^ cells revealed significant downregulation of the gene encoding E-cadherin (Fig. 2C). Furthermore, expression of genes encoding vimentin, MMP-9, IL-6 and β-catenin was upregulated significantly in PC3^RANKL^ cells. Protein expression analysis revealed that PC3^RANKL^ cells expressed significantly lower levels of E-cadherin than PC3^Wild^ cells (Fig. 2D). By contrast, expression of MMP-9, IL-6, c-MYC and β-catenin was significantly higher in PC3^RANKL^ cells.

PC3^RANKL^ cells showed a significant increase in TOP/FOP luciferase reporter activity (Fig. 2E). Immunoprecipitation of β-catenin was carried out to investigate the signal transduction pathway associated with TCF in PC3^RANKL^ cells (Fig. 2F). PC3^RANKL^ cells overexpressing RANKL showed a significant increase in activation of the MAPK and β-catenin/TCF-4 signalling pathways owing to stronger binding between β-catenin and TCF-4 than in PC3^Wild^ cells. Thus, ectopic overexpression of RANKL may increase EMT and the metastatic properties of PC3 cells via the β-catenin/TCF-4 signalling pathway, suggesting the therapeutic potential of RANKL targeting for prevention of PCa metastasis.

### Therapeutic effects of mRANKL-MT in PC3 cell-inoculated mice

**Figure 3:**
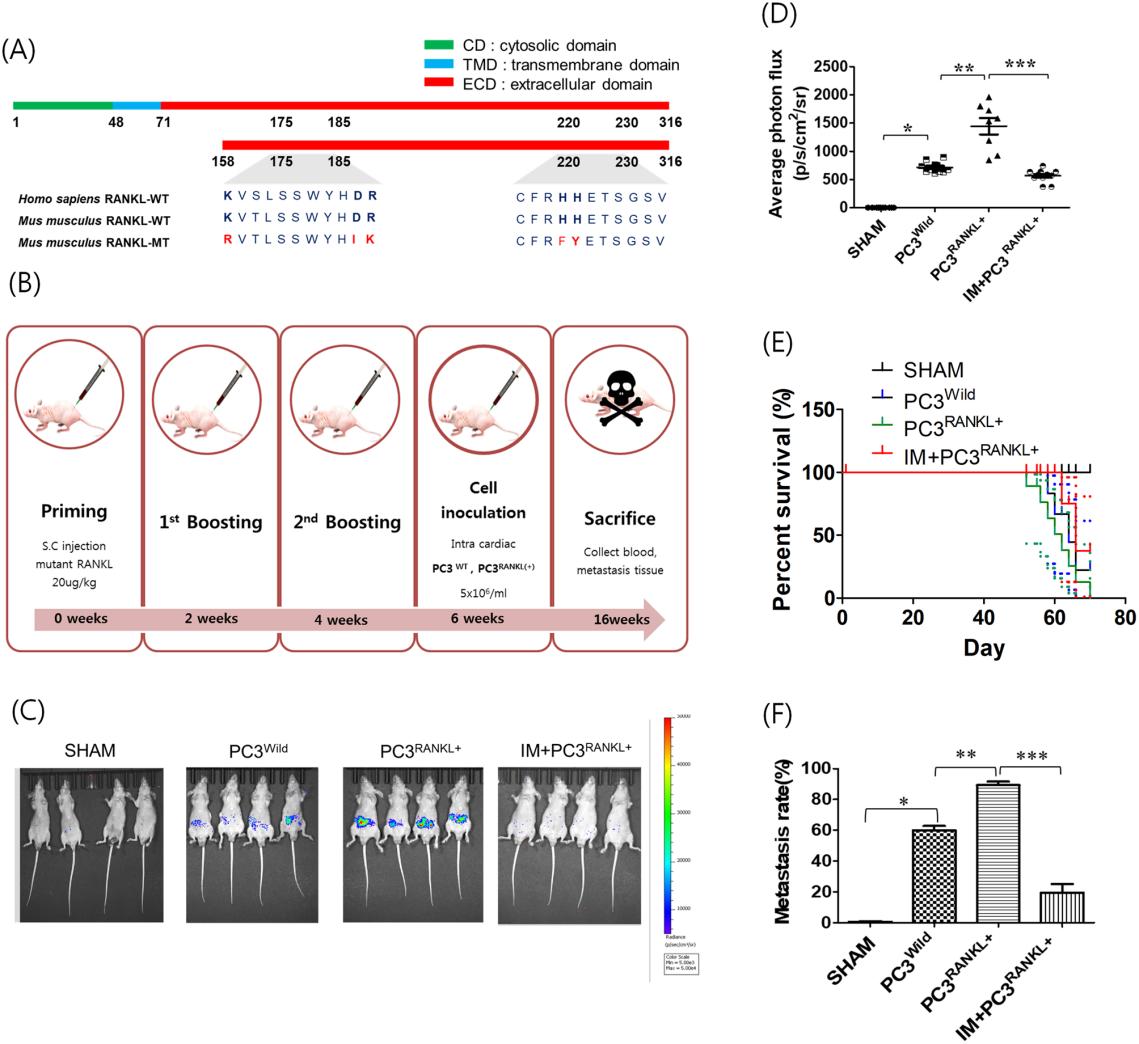
Effects of immunisation with mutant RANKL. (**A**) Comparison of the sequences of hRANKL, mRANKL-WT and mRANKL-MT. (**B**) In vivo experimental flow. Male BALB-c/nu mice (6-weeks-old) were immunised with a subcutaneous injection of test agent (20 μg/kg). (**C**) Tumour growth and metastases were monitored via in vivo bioluminescence imaging of the reporter activity induced by PC3^Wild^, PC3^RANKL+^ and PC3^RANKL+^ + IM treatment. Data per representative mouse are shown. A large hot-spot of bioluminescence was observed in vivo following inoculation of mice with PC3^RANKL+^ cells. Multiple localised and distant metastases were observed in vivo after the injection of PC3^RANKL+^ cells into the hearts of nude mice. Coloured bars indicate the bioluminescence signal intensity (photon/s/cm^2^/steradian). (**D**) The bioluminescence photon flux that appeared after inoculation of cells into mice is shown graphically. Data from related graphs are displayed as the mean ± SD. (**E**) Survival and (**F**) metastasis rate in mice from the SHAM, PC3^Wild^, PC3^RANKL+^ and PC3^RANKL+^ + IM groups.

Alignment of the mRNA sequence of mRANKL-MT with that of mRANKL-WT identified a region that could be amplified using selected primers. The recombinant mRANKL-WT sequence encoded the full-length 158 amino acid target region, which includes residues 158 to 316 (Fig. 3A). To create point mutations, Lys180, Asp189-Arg190 and His223-His224 were transformed to Arg180, Ile189-Lys190 and Phe223-Tyr224, respectively. The resulting hRANKL, mRANKL-WT and mRANKL-MT molecules had similar molecular weights (Supp. 3). Male BALB-c/nu mice were injected subcutaneously with hRANKL, mRANKL-WT, or mRANKL-MT (100 μg/kg; three times every 2 weeks). After immunisation, 1 × 10^6^ PC3^Wild^ or PC3^RANKL+^ cells were injected into the left ventricle of Sham or immunised mice. Serum and tumour-bearing tissues were collected after 16 weeks (Fig. 3B).

To observe bone metastasis, luciferase activity in tumour-bearing tissues of Sham, PC3^Wild^, PC3^RANKL+^ and PC3^RANKL+^ + IM mice was detected by IVIS. In PC3^RANKL+^ mice, large and strong bioluminescence spots were detected throughout the body at 16 weeks post-cancer cell injection (Fig. 3C). However, no bioluminescence signals were detected in PC3^RANKL+^ + IM mice. The photon flux values were significantly higher in PC3^RANKL+^ mice than in PC3^RANKL+^ + IM mice (Fig. 3D).

Survival rate analysis revealed a significant decrease in the survival of animals in the PC3^RANKL+^ groups compared with that of animals from the PC3^Wild^ group. The survival rate was higher in the PC3^RANKL+^ + IM group than that in the PC3^RANKL+^ group (Fig. 3E). The metastasis rate in the PC3^RANKL+^ group was higher than that in the PC3^Wild^ group; however, that in the PC3^RANKL+^ + IM group was significantly less than that in the PC3^RANKL+^ group (Fig. 3F).

### Therapeutic effects of anti-RANKL antibodies induced by RANKL immunisation

**Figure 4:**
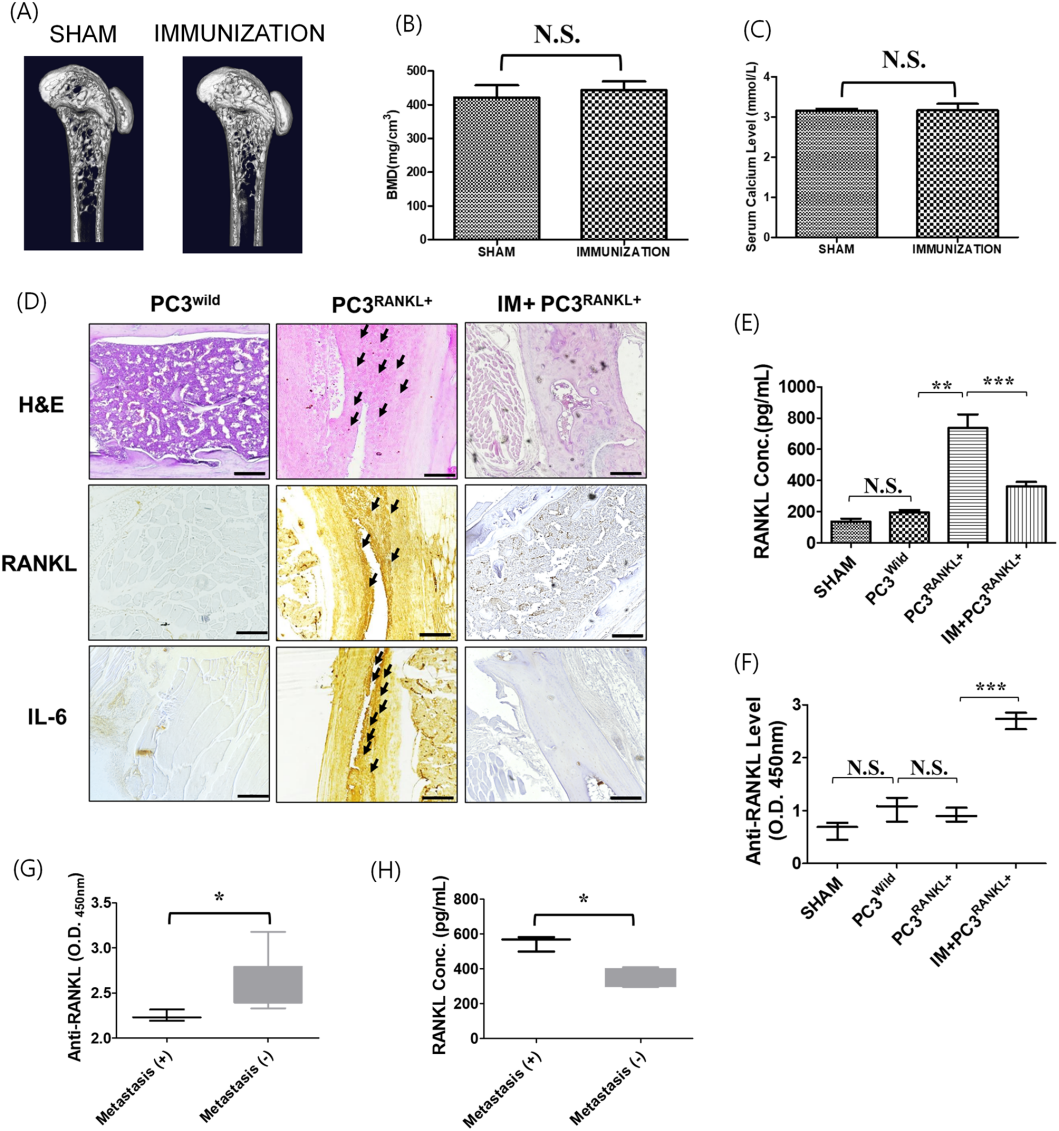
Effects of immunisation with mutant RANKL on PC3-the innoculated metastatic model. (**A**) Three-dimensional micro-CT images revealed the trabecular bone architecture of the volume of interest in SHAM and mRANKL-immunized (IMMUNIZATION) mouse femurs (n = 10 images taken in total, one image for each mouse). (**B**) Bone mineral density (BMD) and (**C**) Serum calcium Level are shown. No significant differences were observed between two groups. (N.S.) (**D**) representative H&E stained and immunostained images of RANKL and IL-6 in the PC3^Wild^, PC3^RANKL+^ and PC3^RANKL+^ + IM groups. The metastatic tumor cells or immuno-positive cells were indicated by black arrows. Magnification: × 200. Scale bar = 100 μm. (**E**) Concentration of RANKL in mouse serum. The mean ± SD values were obtained by densitometry, as shown in the analysis. Significant differences were observed at *p < 0.05 and **p < 0.01 vs. control. (**F**) Serum samples from mice were obtained after immunisation. Anti-RANKL values in PC3^Wild^, PC3^RANKL+^ and PC3^RANKL+^ + IM groups. The mean ± SD values were obtained by densitometry, as shown in the analysis. Significant differences were observed at *p < 0.05 and **p < 0.01 vs. the control. (**G**) Anti-RANKL values and (H) RANKL concentration in the serum of RANKL^-^immunized mice with metastasis (+) or metastasis (−). Bar graphs show the mean ± standard deviation (SD). Significant differences were observed at *p < 0.05, metastasis (+) vs. no metastasis (−).

To demonstrate the effect of RANKL immunization on bone resorption, we examined 3D images in the trabecular bone architecture of the distal femur and serum calcium level. No significant changes in trabecular bones were observed in immunized mice (Fig. 4A), as evident from BMD (Fig. 4B). In addition, there is no significant differences between Sham and immunized mice on serum calcium level (Fig. 4C).

To examine the histological characteristics of metastatic tumour-bearing tissues, metastatic lesions from each mouse were stained with haematoxylin and eosin Y (H&E). As shown in Fig. 4D, gross examination of the excised tibiae from PC3^RANKL+^ mice revealed a tumour mass in the primary spongiosum (trabecular epiphysis) and bone marrow cells; this was not observed in PC3^RANKL+^ + IM mice. In particular, expression of IL-6 and RANKL increased markedly in the trabecular epiphysis region of bones from PC3^RANKL+^ mice, but were undetectable in PC3^RANKL+^ + IM mice.

Next, we investigated whether mRANKL-MT induces production of anti-RANKL antibodies. The concentration of RANKL (Fig. 4E) was highest in PC3^RANKL+^, and production of antibodies (Fig. 4F) was highest in PC3^RANKL+^ + IM mice. Also, we detected and measured anti-RANKL antibody levels in the PC3^RANKL +^ + IM group with bone metastasis to investigate generation of anti-RANKL antibodies after immunisation with mRANKL-MT. The anti-RANKL by mRANKL-MT immunization were detected in entire immunized mice (Supple 4), anti-RANKL titer in mice without bone metastasis was significantly higher than that in mice with bone metastasis (Fig. 4G). Also, serum RANKL levels in the PC3^RANKL+^ + IM group without metastasis were significantly lower than those in mice with metastasis (Fig. 4H). These observations suggest that anti-RANKL antibodies generated by RANKL immunisation suppress metastasis of PCa cells.

### Effect of immunisation on EMT and metastasis

**Figure 5:**
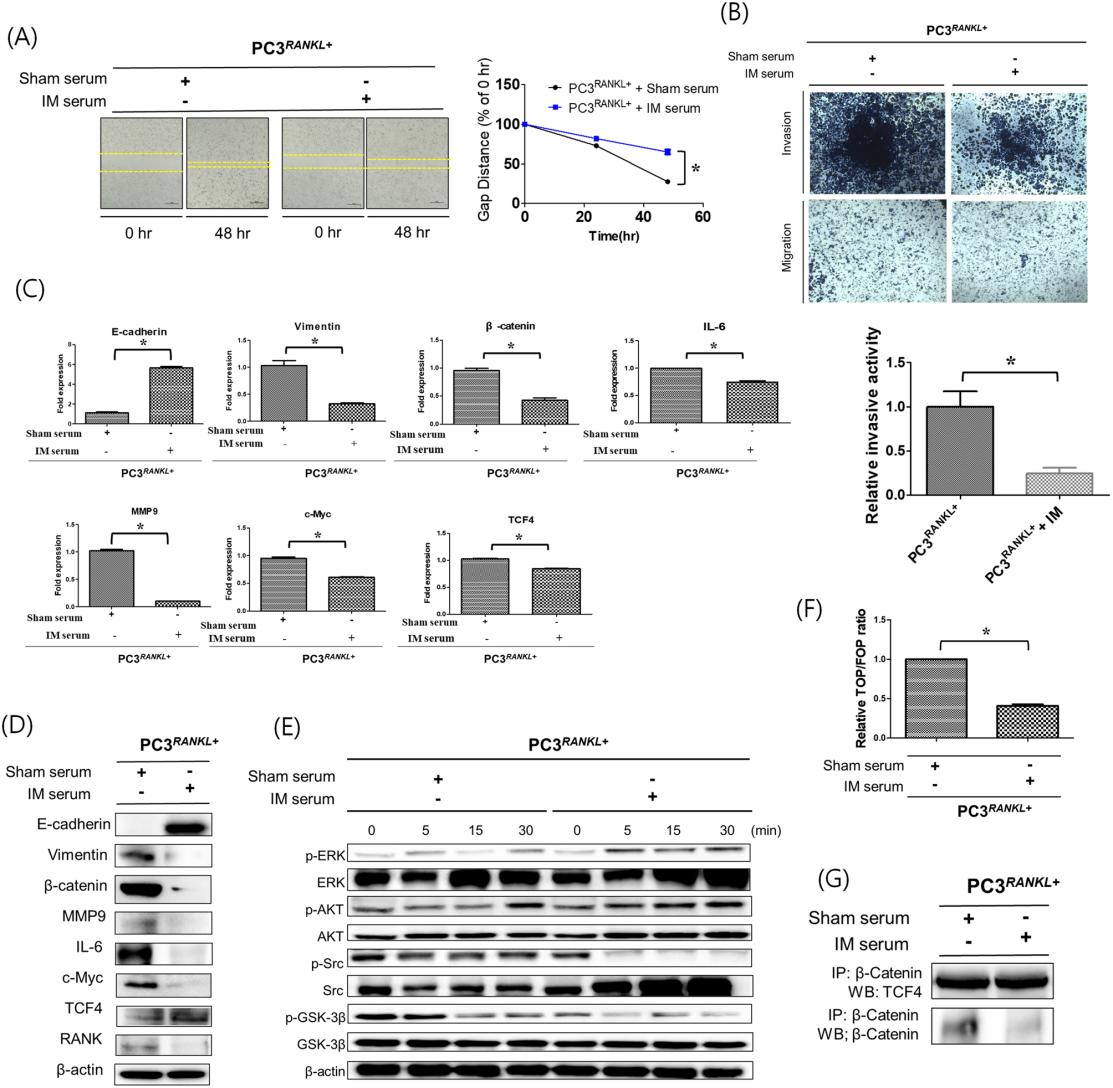
EMT and metastatic properties of PC3 cells treated with immune sera. (**A**) Cell migration assays were performed to compare the wound healing capacity of immune serum-treated PC3^RANKL+^ cells and untreated cells. Immune serum treatment decreased the migration capacity of PC3 cells, as evident from the increase in the wound healing gap at 48 h. Magnification, × 100; scale bar, 100 μm. Data from related graphs are displayed as the mean ± SD. (**B**) Transwell invasion assays were performed to compare the invasiveness of immunised serum-treated PC3^RANKL+^ and untreated cells. Serum-treated PC3^RANKL+^ cells showed a significant decrease in invasiveness. Magnification, × 100; scale bar, 100 μm. Data in the associated graphs are expressed as the mean ± SD. (**C**) Real-time quantitative polymerase chain reaction for the analysis of EMT and metastasis markers in PC3^RANKL+^ cells. β-Actin was used as a loading control. Significant differences were observed at *p < 0.05, compared with the control. (**D**) Expression of E-cadherin, Vimentin, β-catenin, MMP-9, IL-6, c-MYC, TCF-4 and β-actin in immune serum-treated PC3^RANKL+^ cells, as measured by western blotting. β-Actin was used as a loading control. Similar results were obtained in three independent experiments. (**E**) Western blot analysis of MAPK phosphorylation levels in serum-treated PC3^RANKL+^ cells. Similar results were obtained in three independent experiments. (**F**) TOP/FOP luciferase reporter assays in immune serum-treated PC3^RANKL+^ cells. Significant differences were observed at *p < 0.05 *vs.* the control. (**G**) Co-immunoprecipitation of β-catenin and TCF-4 from PC3^RANKL+^ and immune serum-treated PC3^RANKL+^ cells. Each blot was obtained under the same experimental conditions.

To investigate the effects of mRANKL-MT on PCa metastasis, sera obtained from immunised mice were used to treat RANKL-overexpressing PC3 cells. The results of cell migration assays showed that wound healing was inhibited significantly in PC3^RANKL+^ cells treated with immune serum (Fig. 5A). In addition, the invasive ability of PC3^RANKL+^ cells declined following treatment with immune sera (Fig. 5B).

Analysis of mRNA encoding EMT- and metastasis-related factors in PC3^RANKL+^ cells treated with immune sera revealed significant upregulation of E-cadherin expression (Fig. 5C). By contrast, expression of vimentin, MMP-9, IL-6 and β-catenin was downregulated significantly in immune serum-treated PC3^RANKL+^ cells. Protein expression analysis showed that E-cadherin expression was higher in immune serum-treated PC3^RANKL+^ cells than in control serum-treated PC3^RANKL+^ cells (Fig. 5D). By contrast, expression of MMP-9, IL-6 and β-catenin was significantly lower in immune serum-treated PC3^RANKL+^ cells, as was expression of c-MYC.

In addition, protein expression of phosphorylated ERK, AKT, SRC and GSK-3B to investigate the effect of immune serum on MAPK and Wnt signaling in PC3^RANKL+^ cells (Fig. 5E). Cells treated with immune serum showed a significant and time-dependent increase in GSK-3B phosphorylation and a time-dependent decrease in SRC phosphorylation. The luciferase activity of the TOP/FOP reporter in immunised serum-treated PC3^RANKL+^ cells fell significantly (Fig. 5F). Finally, immunoprecipitation analysis revealed a significant decrease in binding between β-catenin and TCF-4 in cells treated with immune serum (Fig. 5G). These results indicate that the EMT and metastatic properties of PC3^RANKL+^ cells were inhibited by treatment with immune serum.

## Discussion

PCa, which is common among men in the western world, is associated with high mortality and morbidity with respect to advanced metastasis to the bone. Evidence suggests that the RANKL signalling cascade plays a key role in proliferation, metastasis, migration and invasion of PCa^6^. The RANKL–RANK interaction plays a pivotal role in PCa metastasis; indeed, RANKL expression induces osteoclast hyperplasia and bone destruction during PCa metastasis^26^. RANKL activates RANK directly on tumour cells, as evidenced by dysregulation of several biochemical signalling pathways in PCa cells.

High expression of RANKL facilitates PCa metastasis, an idea consistent with previous studies showing that signalling through the RANK/RANKL axis is related to bone metastases of solid tumours^20,27^. Especially, several previous study demonstrated that primary prostate cancer cells expressed the RANK/RANKL genes, which was further elevated in bone metastasis lesions^28–30^. Therefore, denosumab which is a specific human monoclonal antibody against RANKL, was found to be a new therapeutic option is expected to exert its antitumor effect by inhibiting RANKL.

We found that hRANKL-treated or hRANKL-overexpressing PCa cells showed a significant increase in expression of metastasis markers such as IL-6. In particular, in vitro experiments show that RANKL stimulation markedly increases the migration and invasion of PC3 cells, downregulates expression of the epithelial marker E-cadherin and upregulates the mesenchymal marker vimentin. EMT correlates with tumour metastasis and progression, which is consistent with impaired cell–cell adhesion following the loss of E-cadherin expression^31,32^. Furthermore, we show that GSK-3B phosphorylation was reduced significantly following RANKL treatment of PC3 cells due to the effect of RANKL on Src-ERK-AKT signalling. In the present study, Top/Fop luciferase activities and TCF4//β-catenin co-expressions were slight increased after RANKL treatment. As these results, RANKL treatment itself affect directly Src-AKT-GSK-3beta, but affect indirectly Wnt/beta-catenin signalling. Especially, GSK-3Beta is considered to be modulated by Wnt or RANK signal and it could affect the beta-catenin activation. Also, we observed altered expression of β-catenin and TCF-4 in PC3^RANKL+^ cells, which resulted in a highly conserved developmental signalling pathway that includes the major effector protein β-catenin. Wnt signalling is an essential pathway involved in cell development, proliferation and differentiation; indeed, regulatory abnormalities in Wnt signalling are associated with metastasis of many cancers^33^. In particular, RANKL overexpression in PC3 cells led to a significant increase in expression of Wnt3a, suggesting that RANKL is a potential target of Wnt signalling in cancer cells^34^. RANKL plays a fundamental role in osteoclastogenesis by interacting with the RANK receptor on osteoclast progenitors during bone destruction by metastatic breast cancer, thereby driving osteoclast cell lineage commitment, monocyte cell fusion and osteoclast maturation via regulation of NF-κB-mediated gene expression; therefore, we were intrigued to find out whether catabolic Wnt signalling mechanisms exist alongside anabolic Wnt pathways to regulate osteoclast formation in bone. β-Catenin, the critical effector of the Wnt pathway, regulates a number of key processes during development, including proliferation, differentiation and cell fate determination^35^. Normally, β-catenin is localised to the cell adhesion junctions in epithelial cells and its abnormal cytoplasmic/nuclear stabilisation drives uncontrolled transcription of target genes (including c-jun, cyclin D1, c-myc, survivin and MMP-7) that regulate cell proliferation, survival and adhesion^36^. In view of cancer cell fate, it is not surprisingly that overexpression of RANKL by PC3 cells led to increased binding of β-catenin to TCF4 and to increased TOP activity. Regulation of β-catenin is linked to the pathogenesis of a number of human cancers, particularly those with an epithelial cell origin. Supporting its putative role as a Wnt signalling target, we confirmed that RANKL overexpression led to transcriptional activation of β-catenin in PC3 cells.

Over the past decades, it has become clear that the RANK/RANKL axis exerts a broad range of functions during cancer cell fate. In the cancer setting, the RANK-RANKL pathway plays a role in every stage of tumorigenesis. Therefore, inhibition of RANKL by anti-RANKL antibodies is expected to be more far-reaching than simple inhibition of cancer cell activation. Denosumab, a drug used to treat metastatic prostate bone loss, has received FDA approval; this drug inhibits the RANK-RANKL pathway^37^. Denosumab is an effective and safe drug, and it is superior to zoledronic acid in terms of the prevention of skeletal-related events; this was borne out in a combined analysis that included three randomised phase III trials with a similar set-up^38^. These trials included patients with bone metastases due to advanced breast cancer^39^, prostate cancer^40^, other solid tumours or multiple myeloma^41^. However, despite medical and commercial success, passive anti-cytokine drugs such as OPG-Fc and denosumab have several limitations, including high production costs, the need for regular infusion, and a limited half-life^42^, indicating the need for a RANKL vaccine. In comparison with antibodies and other biologics, vaccines are better models for treatment of chronic disease because they are relatively cheap and small doses of protein can have a strong and long-lasting effect^43,44^. Here, we developed a novel vaccine targeting RANKL and examined its efficacy in a murine model of prostate cancer metastasis. To circumvent the problem of the immunogen triggering cytokine activity, mutants of RANKL were generated to prevent its interaction with RANK. A previous study shows that immunisation with mutant RANKL molecules generates anti-RANKL antibodies that block the interaction between RANKL and its receptor in an animal model of osteoporosis, thereby preventing proliferation and differentiation of osteoclasts and improving bone density^45^. Therefore, to block RANKL activation during PCa metastasis, we immunised mice with mRANKL-MT followed by intracardiac injection of PC3 cells. Inhibiting RANKL in animal models of metastases exerts therapeutic effects by inhibiting cancer cell metastasis. Currently, in vivo tumour models that are most commonly used to study the process of cancer metastasis rely on introduction of tumour cells directly into the systemic circulation by injection into the left ventricle of laboratory rodents^46,47^. Thus, we employed a mouse model of PCa metastasis that more accurately reflects the metastatic process of this type of cancer. Studies on the effects of mRANKL-MT in PC3^RANKL+^ mice showed that tumour growth was completely inhibited. Immunisation with mRANKL-MT effectively inhibited metastasis of tumour cells by generating anti-RANKL antibodies.

**Figure 6:**
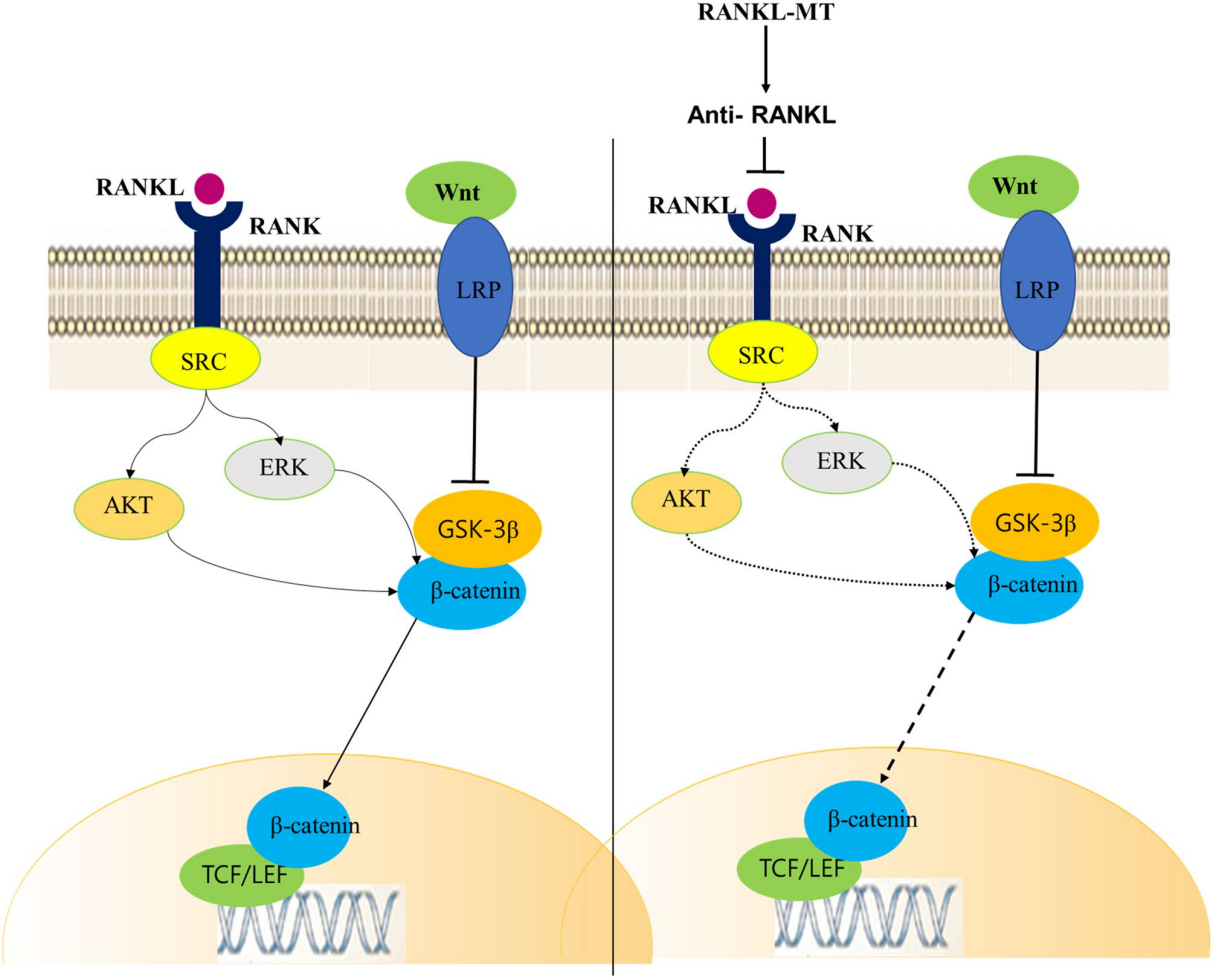
Schematic diagram illustrating anti-RANKL-mediated inhibition of RANK signaling. Activation of RANK by RANKL activates AKT and ERK, thereby liberating the active form of β-catenin. Generation of anti-RANKL antibodies by mRANKL-MT inactivates RANKL, thereby downregulating AKT/ERK activity and preventing downstream of β-catenin signaling.

To further confirm the action of RANKL immunisation, we assessed the effects of immune serum from immunised mice on PCa cells. Anti-RANKL antibodies blocked the RANKL-mediated chemotaxis of tumour cells. Furthermore, anti-RANKL antibodies inactivated RANKL on tumour cells directly. Treatment of RANKL-overexpressing PC3 cells with immune serum almost entirely abolished cancer cell migration and invasion. Wu et al. showed that a recombinant inactive RANKL vaccine (Y234pNO2Phe) induced high antibody titer and protected mice from collagen-induced arthritis by inhibiting osteoclast function and by preventing bone erosion^48^. However, no study has reported that these types of RANKL vaccine have been used to inhibit cancer metastasis. In addition, we found that EMT and metastasis-related genes were downregulated following treatment of RANKL-overexpressing PC3 cells with immune serum. The antisera obtained from mice immunised with mRANKL-MT almost entirely inhibited the EMT process in RANKL-overexpressing PC3 cells. EMT is characterised by the loss of cell–cell adhesion and by an increase in cell motility; it is a key process in cancer progression and metastasis, making EMT inhibition an attractive therapeutic strategy^49,50^. Deregulation of Wnt/β-catenin signalling is a hallmark of PCa metastasis^51,52^ and β-catenin is a critical end component of the Wnt signalling pathway, which regulates cell growth, apoptosis and migratory behaviour in response to intercellular adhesion molecules^53^. Activation of β-catenin in PCa cells leads to transactivation of Wnt signalling target genes, including cyclin D1, HEF1 and matrix metalloproteinase 9^54^. Also, previous studies show that expression of Wnt-1 and β-catenin is increased in invasive PCa cell lines and in primary prostate cancer specimens^55,56^. In line with these previous reports, we demonstrated that treatment of PC3 cells with immune serum led to a marked decrease in RANK/SRC and GSK-3β signalling and β-catenin/TCF-4 transcription (Fig. 6). β-Catenin forms a cell adhesion complex with E-cadherin, raising the possibility that loss of expression or a change in β-catenin distribution in the cell alters downstream signalling, decreases intercellular adhesion and promotes metastasis. These results suggest that the inhibitory effect of immune serum on PCa cell metastasis may involve suppression of the Wnt/β-catenin signalling pathway.

The present study has several strengths and limitations. One potential advantage of a vaccination approach to RANKL inhibition compared with antibody-based approaches such as denosumab is that patients who discontinue treatments experience rapid increases in bone remodelling and an increased risk of osteolytic fractures during bone metastatic cancer. It is possible, though clearly unproven, that this vaccine approach may not induce rapid high-turnover bone loss, either by causing more durable RANKL inhibition or by allowing a more gradual resumption of remodelling when vaccinations are discontinued. That would be reasonable which vaccine-type approach could be tested for its ability to minimize the risk of high-turnover bone loss through in vitro and in vivo study.

One limitation of present study is a lack of validation of safety or efficacy beyond long-term rodent studies, despite their experimental reality approach. Because generated mutant RANKL is transformed from mouse RANKL, several amino acid sequences are different from human RANKL and the clinical relevance of the present results for human study thus remains unclear. Therefore, it needs to be carried out in human RANKL knock-in mouse model using by human RANKL mutant variant in further study.

Taken together, this study demonstrates the protective role of mRANKL-MT against RANKL-induced PCa in mice. This effect was mediated via induction of a high-titer antibody response and inhibition of EMT and metastatic functions. Our results highlight the potential application of an anti-RANKL vaccine for treatment of metastatic RANKL-induced PCa. Moreover, the results suggest that mutant RANKL could be used as a RANKL vaccine for the prevention and/or treatment of patients with metastatic PCa prostate cancer.

## Methods

### Cell lines and cultures

Human prostate adenocarcinoma luciferase-labelled PC3^luc^ cells were obtained from Professor Park and maintained at 37 °C/5% CO_2_ in Rowell Park Memorial Institute RPMI 1640 medium (Welgene, Gyeongsansi, Korea) supplemented with 10% heat-inactivated foetal bovine serum (GIBCO, Waltham, MA, USA) and a 10% antibiotic solution (Welgene).

### Cloning of hRANKL

The RNA used to clone hRANKL cDNA was extracted from MG63 cells (ATCC CRL-1427) expressing RANKL. The quality of the extracted RNA was verified by agarose gel electrophoresis. The cDNA was prepared using the AccuPower RT PreMix Kit (Bioneer, Daejeon, Korea), according to the manufacturer’s instructions. Amplification and cloning of the hRANKL fragment were carried out in a reaction mixture comprising KOD polymerase buffer, 10 mM dNTPs, 25 mM magnesium chloride (MgCl_2_), 10 μM primers (hRANKL-*Bcl*I: 5′-TGATCAAAGCTTGAAGCTCAGCCTTTTGC-3′ and hRANKL**-Xho**I: 5′-CTCGAGATCTATATCTCGAACTTTAAAAGCCCC-3′), 2.5 U of KOD DNA polymerase (EMD Millipore, Billerica, MA, USA) and 2 μL of the RANKL gene construct (template). The thermal cycling conditions were as follows: initial denaturation at 95 °C for 5 min, followed by 40 cycles of denaturation at 95 °C for 30 s, annealing at 55 °C for 30 s and extension at 70 °C for 30 s. The polymerase chain reaction (PCR) product was cloned into the *Bam*HI/*Xho*I sites of a pMX vector (CELL BIOLABS, USA). Sequence analyses were carried out using programs in Vector NTI Advance 9.1.0 (Invitrogen, Carlsbad, CA, USA).

### Retroviral hRANKL transduction

Plat-E cells were seeded at a density of 3 × 10^5^ cells/well in a six-well plate for 24 h and then transiently transfected with hRANKL/pMX using 0.2 μg plasmid and 0.6 μL of the FuGENE HD transfection reagent (Promega, Madison, WI, USA), according to the manufacturer’s protocol. After incubation, the DNA/FuGENE mixture was added drop-wise onto Plat-E cells. Viral supernatants were recovered from the culture medium at 48 h after transfection. Virus-containing supernatants were filtered through 0.45 μm non-pyrogenic filters and supplemented with 10 μg/mL polybrene (Sigma-Aldrich, St. Louis, MO, USA).

### Real-time quantitative PCR (RT-qPCR)

Total RNA was extracted from PCa cells using Trizol (Invitrogen) and 1 μg was used for RT-qPCR along with oligo-dT primers (10 μg) and dNTPs (10 mM). Next, qRT-PCR was performed to analyse cDNA using SYBR Green SuperMix (Bio-Rad Laboratories, Inc. Hercules, CA, USA) on a CFX Connect Real-Time System (Bio-Rad). All target gene primers were purchased from Bioneer Co. (Daejeon, Korea) and the cDNA was amplified using the following primer sets:

E-cadherin (h): 5′-TGGAGGAATTCTTGCTTTGC-3′ (forward) and 5′-TGGAGGAATTCTTTTGC-3′ (reverse); vimentin (h): 5′-GACGCCATCAACACCGAGTT-3′ (forward) and 5′-GACGCCATC AACACCGAGTT-3′ (reverse); β-catenin (h): 5′-ACAAACTGTTTTGAAAATCCA-3′ (forward) and 5′-CGAGTCATTGCATACTGTCC-3′ (reverse); MMP-9 (h): 5′-TCCAGTACCAAGACAAAG-3′ (forward) and 5′-TTGCACTGCACGGTTGAA-3′ (reverse); RANK (h): 5′-CAAATGCAGACCCTGGA CCA-3′ (forward) and 5′-AAACGCCAAAGATGATGGCA-3′ (reverse); RANKL (h), 5′-CCTGTAT GCCAACATTTGCTTTC-3′ (forward) and 5′-TTCCTCTCCAGACCGTAACTTAAA-3′ (reverse); IL -6 (h): 5′-AGCAAAGAGGCACTGGCAGA-3′ (forward) and 5′-GTACTCATCTGCACAGCTCTGG C-3′ (reverse); TCF-4 (h): 5′-GCTCAGGGTATGGAACCGGC-3′ (forward) and 5′-CCCTGTAGTC CTGGTGGCATG-3′ (reverse); c-MYC (h): 5′-CCTGGTGCTCCATGAGGAGAC-3′ (forward) and 5′-AGACTCTGACCTTTTGCCAGG-3′ (reverse); and glyceraldehyde 3-phosphate dehydrogenase (GAPDH): 5′-TCAAGAAGGTGGTGAAGCAG-3′ (forward) and 5′-AGTGGGAGTTGCTGTTGAAG T-3′ (reverse). Values on the vertical axis represent 2(− ΔCt); ΔCt is the discrepancy between the target gene Ct and GAPDH Ct.

### Western blot analysis

The cells were washed twice with phosphate-buffered saline (PBS; pH 7.4) and total proteins were extracted using radio immunoprecipitation assay buffer supplemented with 1% protease inhibitors, phosphatase inhibitors and phenylmethylsulfonyl fluoride (PMSF). The protein concentration was measured using the BCA Protein Assay Kit (Thermo Pierce, Waltham, MA, USA). The membrane was blocked with a solution containing 5% skim milk in TBS-T for 30 min and then washed in TBS-T. The membrane was incubated for overnight at 4 °C with the following primary antibodies: E-cadherin (sc-7870, Santa Cruz Biotechnology, Inc., Dallas, TX, USA), β-catenin (#29822 94, Millipore, Burlington, MA, USA), MMP-9 (#13667, Cell Signaling Technology, Denvers, MA, USA), IL-6 (#12153, Cell Signaling Technology), c-MYC (9E10, Santa Cruz Biotechnology), TCF-4 (#2565, Cell Signaling Technology), RANK (#4845, Cell Signaling Technology), P-ERK (#9101, Cell Signaling Technology), ERK (#9102, Cell Signaling Technology), GAPDH (#2118, Cell Signaling Technology), P-AKT (#9271, Cell Signaling Technology), AKT (#9272, Cell Signaling Technology), P-SRC (#2105, Cell Signaling Technology), SRC (#2108, Cell Signaling), GSK-3B (#9315, Cell Signaling Technology) and P-GSK-3B (#9336, Cell Signaling Technology). Horseradish peroxidase (HRP)-conjugated AffiniPure goat anti-rabbit IgG (H + L) and HRP-conjugated AffiniPure goat anti-mouse IgG (H + L) were obtained from Proteintech Group, Inc (Chicago, IL, USA) and used as secondary antibodies. And then, the membrane was washed in TBST, and protein immunoreactivity was detected using an enhanced chemiluminescence detection kit (Sigma Aldrich Inc., Saint Louis, MO, USA). Finally, the blot images were acquired by chemiluminescence imaging system (Vilber Co., Collégien, France).

### BrdU cell proliferation assay

The BrdU cell proliferation assay was performed by means of BrdU cell proliferation assay kits (EMD Biosciences, Inc., Darmstadt, Germany) according to the manufacturer’s instructions. Briefly, culture medium was removed, 100 mL of fresh culture medium and 20 mL of BrdU labeling solution were added to each well for 12 h. After 12 h of incubation with BrdU, cells were fixed and incubated with anti-BrdU conjugated with peroxidase. Subsequent to substrate addition, the optical density at 450 nm with a reference wavelength of 540 nm (OD450/540) was determined by microplate reader (BioTek Instrument, Winooski, VT, USA).

### Cell migration assay

Cells were seeded in 6-well plates for the cell migration assay. After each treatment, a confluent monolayer was wounded using a 200 μL pipette tip. Images of wound closure were obtained under an inverted microscope after 48 h. The wound area was calculated using NIH ImageJ software.

### Cell invasion assay

A total of 1 × 10^5^ transfected cells were seeded into the top chamber of a 24-well polycarbonate Transwell chamber (8.0 µm pore size; Corning Incorporated, Glendale, AZ, USA) and then treated for 24 h with indicated treatment. After 24 h of incubation, non-invading cells on the top of the membrane were removed by cotton swabs. Invaded cells on the bottom of the membrane were fixed with 4% paraformaldehyde for 10 min, followed by staining with 0.05% crystal violet for 4 h. The cells were taken pictured and all the cells on the entire membrane were counted. The relative invasion activity was calculated after normalization to cell migration.

### Luciferase reporter assay to assess Wnt/β-catenin activity

Cells were seeded into a 24-well plate 24 h prior to transient transfection with either 2 μg of TOP flash or FOP flash reporter plasmid along with 1 μg DNA using Lipofectamine 3000 (Thermo Fisher). The TOPflash luciferase reporter plasmid contains TCF-4-binding sites upstream of the luciferase gene, resulting in luciferase activity in the presence of active Wnt/β-catenin signalling. The FOP flash reporter plasmid, on the other hand, carried mutated TCF-4-binding sites. Total cell extracts were assayed for luciferase activity according to the manufacturer’s instructions (Promega).

### Immunoprecipitation

Cells were lysed in lysis buffer (20 mM Tris–HCl pH 7.6–8.0, 100 mM sodium chloride NaCl, 300 mM sucrose, 3 mM MgCl_2_ [buffer A]; and 20 mM Tris pH 8.0, 100 mM NaCl, 2 mM ethylenediaminetetraacetic acid [buffer B]). Whole cell lysates obtained by centrifugation were incubated with antibodies specific for active β-catenin (Millipore) and TCF-4 (Cell Signaling Technology) (dilution 1:100) and protein A Sepharose beads (Amersham Biosciences) for 2 h at room temperature. The immune complexes were washed three times using wash buffer and examined by western blotting.

### Site-directed mutagenesis and production and purification of mRANKL-MT

The RNA used for the cloning of RANKL cDNA was extracted from MC3T3-E1 cells (Korean Cell Line Bank, Seoul, Korea) expressing RANKL. The quality of the extracted RNA was verified by agarose gel electrophoresis and cDNA was prepared using the AccuPower RT PreMix Kit (Bioneer), according to the manufacturer’s instructions. Amplification and cloning of the RANKL fragment were carried out in a reaction mixture comprising KOD polymerase buffer, 10 mM dNTPs, 25 mM MgCl_2_, 10 μM primers (mRANKL-*Nde*I: 5′-CATATGAAGCCTGAGGCCCAGCC ATTTGC-3′; mRANKL-*Xho*I: 5′-CTCGAGGTCTATGTCCTGAACTTTGAAAGCC-3′; mRANKL (K180R)-F: 5′-CCCATCGGGTTCCCATCGAGTCACTCTGTCCTCTTG-3′; mRANKL (K180R)-R: 5′-CAAGAGGACAGAGTGACTCGATGGGAACCCGATGGG-3′; mRANKL (D189I, R190K)-F: 5′-CTCTTGGTACCACATCAAGGGCTGGGCCAAGAT-3′; mRANKL (D189I, R190K)-R: 5′-ATC TTGGCCCAGCCCTTGATGTGGTACCAAGAG-3′; mRANKL-MT (H223F, H224Y)-F: 5′-AA CA TTTGCTTTCGGTTTTATGAAACATCGGGAAGCG-3′; or mRANKL-MT (H223F, H224Y)-R: 5′-CGCTTCCCGATGTTTCATAAAACCGAAAGCAAATGTT-3′), 2.5 U of KOD DNA polymerase (EMD Millipore, Billerica, MA, USA) and 2 μL of RANKL gene construct as the template.

The thermal cycling conditions were as follows: initial denaturation at 95 °C for 5 min, followed by 40 cycles of denaturation at 95 °C for 30 s, annealing at 55 °C for 30 s and extension at 70 °C for 30 s. The PCR product obtained was cloned into the *Nde*I/*Xho*I site of the GST-30a vector (Novagen, Madison, WI, USA). Mutations at positions 180, 189–190 and 223–224 were introduced using megaprimers^45^. The PCR product was transformed into *Escherichia coli* BL21-CodonPlus (DE3)-RIPL (Novagen) by electroporation (5 ms, 12.5 kV/cm) and the transformed cells were cultivated in Luria–Bertani broth containing kanamycin (50 μg/mL, T&I, Daejeon, Korea). Plasmids were purified using the QIAprep Spin Miniprep Kit (Qiagen, Valencia, CA, USA). The cloned product was confirmed by sequencing. All sequence analyses were carried out using programs in Vector NTI Advance 9.1.0 (Invitrogen). The recombinant plasmid carrying mRANKL-MT was expressed from a single *E. coli* BL21-CodonPlus (DE3)-RIPL colony using previously described methods^45^.

### Purification of mRANKL-MT

*E. coli* cells expressing mRANKL-MT were cultivated in 1 L of an auto-induction medium supplemented with kanamycin (50 μg/mL), as previously described^42^. After centrifugation at 6000×*g* for 20 min at 4 °C, the pelleted cells were resuspended in 10 mL of lysis buffer (20 mM sodium phosphate, 500 mM NaCl, 10 mM imidazole, pH 7.4) supplemented with 0.1 mg/mL lysozyme and 0.1 mM PMSF.

Glycerol (20% v/v; CARLO ERBA, France) was added to the cell suspension and the cells were sonicated and centrifuged at 15,000×*g* for 10 min at 4 °C. The supernatants were passed through 0.2 μm paper filters and applied to Ni^2+^-affinity chromatography HisTrap FF columns (1 mL; GE Healthcare Life Science, Piscataway, NJ, USA) equilibrated with binding buffer (20 mM sodium phosphate, 500 mM NaCl, 10 mM imidazole, 5 mM dithiothreitol, pH 7.4). The columns were subsequently washed using binding buffer supplemented with 20 mM imidazole.

After washing, bound protein was eluted using elution buffer (Qiagen). The eluted protein was dialysed against a dialysis buffer (20% v/v glycerol in PBS) in a 10,000 MW Slide-A-Lyzer Dialysis cassette (Thermo Fisher Scientific). The purified protein was vacuum concentrated (Savant Instruments, Holbrook, NY, USA) and analysed by sodium dodecyl sulphate polyacrylamide gel electrophoresis (SDS-PAGE). Protein concentrations were calculated using the Bradford assay. For endotoxin removal, an additional washing step was introduced after the initial wash for chromatography.

### Animal study

The animal experimental protocol was approved by the Institutional Animal Care and Use Committee, Chosun University, Gwangju, Korea (CIACUC2019-A0015). The study was carried out in compliance with the ARRIVE guidelines. All experiments were performed in accordance with relevant guidelines and regulations. Five-week-old male athymic nude mice (BALB-c/nu, Orient Bio Co. LTD, Seoul, Korea) were used to generate a xenograft model by intracardiac injection of PC3^Wild^, PC3^+RANKL^ (RANKL overexpression), or PC3^+RANKL^ + IM (immunisation) cells. Following immunisation, mice were divided into an immunisation group and a non-immunisation group. The Sham group was immunised by a subcutaneous injection of PBS, while the immunisation group was injected subcutaneously with mRANKL-MT (100 μg/kg three times every 2 weeks). Mouse sera and tissue samples were collected according to the indicated schedule.

### In vivo bioluminescence measurement

Tumour-bearing tissues were subjected to in vivo bioluminescence imaging using a Living Image 4.5.4 IVIS Imaging System (Perkin Elmer). For luciferase imaging, D-luciferin (Promega) was injected intraperitoneally before imaging. Quantitative detection of luciferase was performed as follows: regions of interest (ROIs) were drawn to capture detected fluorescence, and auto-regions ROIs were used to precisely outline the target region.

### Micro-CT imaging data acquisition

Micro-CT scanning for the distal femur was distally initiated at the level of growth plate using a Quantum GX (PerkinElmer, Hopkinton, MA, USA) micro-CT imaging system located at the Korea Basic Science Institute in Gwangju, Korea. The scanning X-ray source was set to 88 mA and 90 kV, with a 10 mm field of view (scanning time, 4 min; voxel size, 20 μm). The 3D architecture images were acquired using 3D Viewer commercial software included with the Quantum GX system. The 3D images were obtained at 4.5 μm resolution. After scanning, the bone structure parameters were analyzed with Analyze 12.0 software (Analyze Direct, Overland Park, KS, USA) using the ROI tool. Femur bone mineral density (BMD) was estimated using hydroxyapatite (HA) Phantom (QRM-MicroCT-HA, Quality Assurance in Radiology and Medicine GmbH, Germany) scanning using the same parameters. Parameter values are shown as mean ± standard deviation (SD).

### Quantitative analysis of RANKL and calcium level in serum

The amount of RANKL in mouse serum was measured using a commercially available enzyme-linked immunosorbent assay (ELISA) kit (R&D Systems, USA), while colorimetric assays were used to assess calcium (Biovision, San Francisco, CA, USA) according to the manufacturer’s protocol. Absorbance was measured in a colorimetric microplate reader (BioTek) at 450 nm.

### Immunoreactivity blot

The mRANKL-WT and mRANKL-MT were separated by SDS-PAGE and electroblotted onto nitrocellulose membranes (Bio-Rad). Membranes were blocked with 5% (wt/vol) nonfat milk powder in TBST [10 mM Tris (pH 7.5), 150 Mm NaCl, 0.1% (vol/vol) Tween 20] and probed with mouse serum as primary antibodies in the blocking solution. The membranes were washed three times with Tris-buffered saline. Horseradish peroxidase-conjugated secondary antibodies were diluted 1:5000 in 1% (wt/vol) nonfat milk powder in TBST. The membranes were developed using the ECL system (Amersham Pharmacia Biotech).

### Histological analysis in tumor bearing mice

Tumor bearing tissues were dissected, immersed in 4% formaldehyde, and decalcified in 7% EDTA with 0.5% paraformaldehyde for 40 days before processing. To analyze longitudinal sections of distal femurs, decalcified tissues were paraffin-embedded and 2–3-μm-thick sections were cut, mounted on glass slides, and rehydrated using graded alcohol. The tissue sections were stained with hematoxylin/eosin (Shandon Varistain 24-4, Histocom, Vienna, Austria), and images were acquired using an ECLIPSE Ts2R inverted microscope (Nikon).

Paraffin sections were deparaffinized in three xylene washes and rehydrated in graded ethanol solutions. For antigen retrieval, the slides were placed in 0.01 M citrate buffer (pH = 6.0) and heated in a steamer for 30 min. Endogenous peroxidases were quenched by incubating the samples with 3% hydrogen peroxide for 20 min at room temperature. The sections were incubated overnight at 4 °C using 1:50 anti-IL-6 (Santa‑Cruz Biotechnology Inc.) or anti-RANKL (Santa‑Cruz Biotechnology Inc.). Sections were then incubated for 30 min with biotinylated secondary antibody (LSAB system HRP kit; Dako Cytomation, Glostrup, Denmark), rinsed in PBS, and incubated for 30 min with a streptavidin‑peroxidase conjugate (LSAB; DakoCytomation). The reaction was developed for 5 min using 3,30‑diaminobenzidine tetrahydrochloride (Sigma‑Aldrich). The slides were counterstained in hematoxylin, dehydrated, and coverslipped. Negative and positive controls were simultaneously analyzed. The positive controls were mammary tissues. The slides were imaged using an inverted microscope (Nikon).

### Measurement of anti-RANKL antibody titers

Serum samples obtained from immunised mice were serially diluted with PBS containing 0.02% sodium azide and 2% bovine serum albumin (BSA), and then applied to ELISA plates (Sigma-Aldrich) coated with mouse recombinant tumour necrosis factor ligand superfamily member 11 (TNFSF11; 10 μg/mL, R&D Systems). Reactivity of serum antibodies to the target protein was determined using an HRP-conjugated goat anti-mouse IgG secondary antibody (Thermo Fisher Scientific) at a dilution of 1/1000 in PBS/0.02% sodium azide/2% BSA. After development with 1,2- phenylenediamine dihydrochloride (0.4 mg/mL in 0.066 M disodium phosphate, 0.035 M citric acid and 0.01% hydrogen peroxide), absorbance was measured in an ELISA plate reader at 450 nm.

### Statistical analysis

Data are expressed as the mean ± standard deviation (SD) from three independent experiments. GraphPad Prism version 6.0 software for windows was used to analyse in vitro and in vivo data*.* Statistical significance for pairwise comparison was evaluated using an unpaired *t*-test or one-way analysis of variance (ANOVA) with Tukey’s post-hoc test. Results were considered significant at *p < 0.05.

### Ethics approval

The animal experimental protocol was approved by the Institutional Animal Care and Use Committee, Chosun University, Gwangju, Korea (CIACUC2019-A0015).

## Supplementary Information

Supplementary Information.

## Abbreviations

PCa: Prostate cancer
NF-κB: Nuclear factor kappa-light-chain-enhancer of activated B cells
RANKL: Receptor activator of NF‐kB ligand
EMT: Epithelial–mesenchymal transition
TCF-4: Transcription factor 4
TRANCE: Tumour necrosis factor-related activation-induced cytokine
OPGL: Osteoprotegerin ligand
ODF: Osteoclastic differentiation factor
GSK-3: Glycogen synthase kinase-3
mRANKL-MT: Mouse RANKL mutant
IL-6: Interleukin-6
RPMI: Roswell Park Memorial Institute
FBS: Fetal bovine serum
HRP: Horseradish peroxidase
PCR: Polymerase chain reaction
IHC: Immunohistochemistry
