# Supplementary material for: RANKL immunisation inhibits prostate cancer metastasis by modulating EMT through a RANKL-dependent pathway
Source: Sci Rep. 2021 Jun 9;11:12186. doi: 10.1038/s41598-021-91721-2 (PMC8190078; doi:10.1038/s41598-021-91721-2)

RANKL immunisation inhibits prostate cancer metastasis by modulating EMT through a RANKL-dependent pathway

Mineon Park[1,2,*], Yong Jin Cho[1,2,*], Bora Kim[1,2], Young Jong Ko[1,2], Yuria Jang[1,2], Yeon Hee Moon[3], Hoon Hyun[4], Wonbong Lim[1,2,5†]

Affiliations

[1]Laboratory of Orthopaedic Research, Chosun University Hospital, Dong-Gu, Gwangju, 61452, Republic of Korea

[2]Department of Orthopaedic Surgery, Chosun University Hospital, Dong-Gu, Gwangju, 61452, Republic of Korea

[3]Department of Dental Hygiene, Chodang University, Muan County, Jeollanam-do, 58530, Republic of Korea

[4]Department of Biomedical Sciences Chonnam National University Medical School, Gwangju 61469, Republic of Korea

[5]Department of Premedical Science, College of Medicine, Chosun University, Dong-Gu, Gwangju 61452, Republic of Korea

*These authors contributed equally to this work.

[†]Correspondence to: Wonbong Lim, Department of Premedical Sciences, College of Medicine, Chosun University, Dong-Gu, Gwangju, 501-759, Korea;

E-mail: **wonbong@chosun.ac.kr**

Phone: +82 62-230-6193; Fax: +82 62-226-3379;

Contact info

Mineon Park parkminun@hanmail.net

Yong Jin Cho choisidoru@chosun.ac.kr

Bora Kim kbr85025@naver.com

Young Jong Ko yeasts@hanmail.net

Yuria Jang youria0526@nate.com

Yeon Hee Moon yhmon@chodang.ac.kr

Hoon Hyun hhyun@jnu.ac.kr

Wonbong Lim wonbong@chosun.ac.kr

Acknowledgements

This study was supported by research funding from Chosun University (awarded in 2020).

Supple. 1

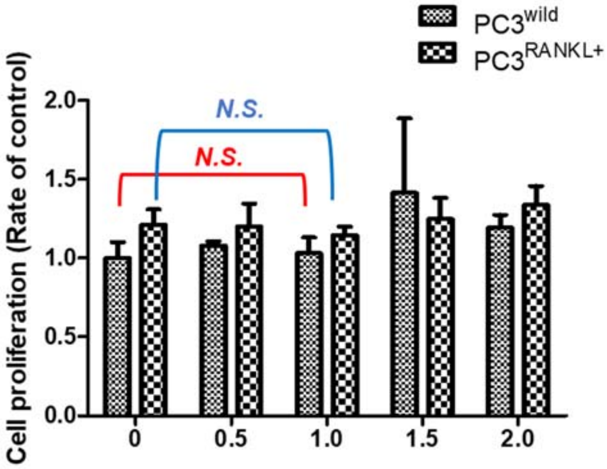

Supple 1. Cell proliferation by BrDu assay on hRANKL treated PC3^{wild} and PC3^{RANKL+} in dose dependent manners. Data are expressed as means ± SD with three independent experiment. No significant differences were detected in compared with 0μg *h*RANKL and 1μg , respectively.

Supple.2

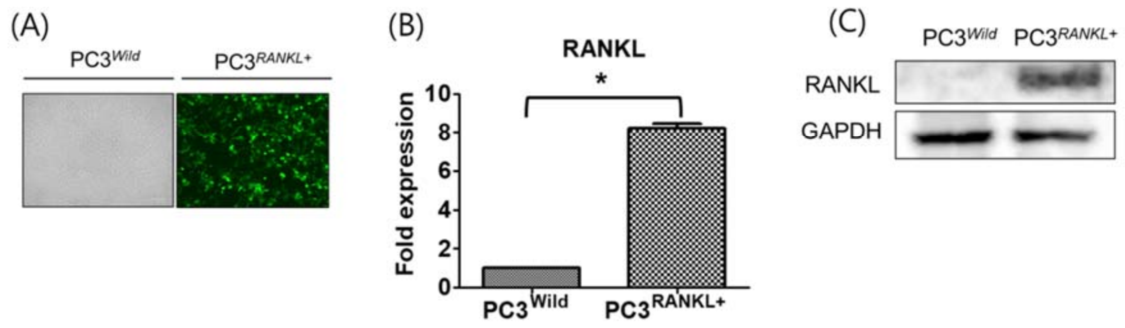

Supple 2. Overexpression of RANKL in PC3. (A) GFP-expressing PC3^{RANKL+} cells were monitored by fluorescence microscopy. Magnification, 100×; scale bar, 100 µm. (B) Reverse-transcription quantitative polymerase chain reaction analysis of *RANKL* mRNA expression in PC3^{Wild} and PC3^{RANKL+} cells. *GAPDH* was used as a loading control. Significant differences were observed at *$p < 0.05$, compared with the control. (C) Western blot analysis of RANKL expression in PC3^{Wild} and PC3^{RANKL+} cells. GAPDH was used as a loading control.

Supple.3

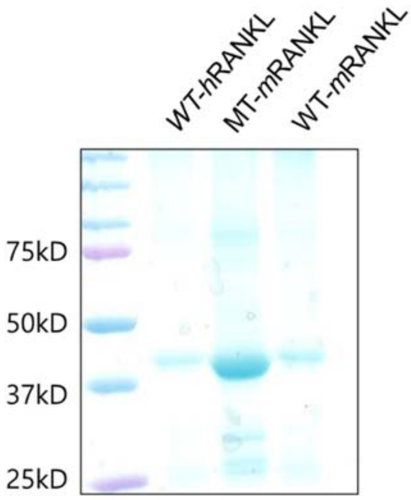

Supple 3. Amplification and cloning of cDNA encoding the active fragments of RANKL.

The molecular sizes of hRANKL, mRANKL-WT and mRANKL-MT were confirmed by SDS-PAGE.

Supple.4

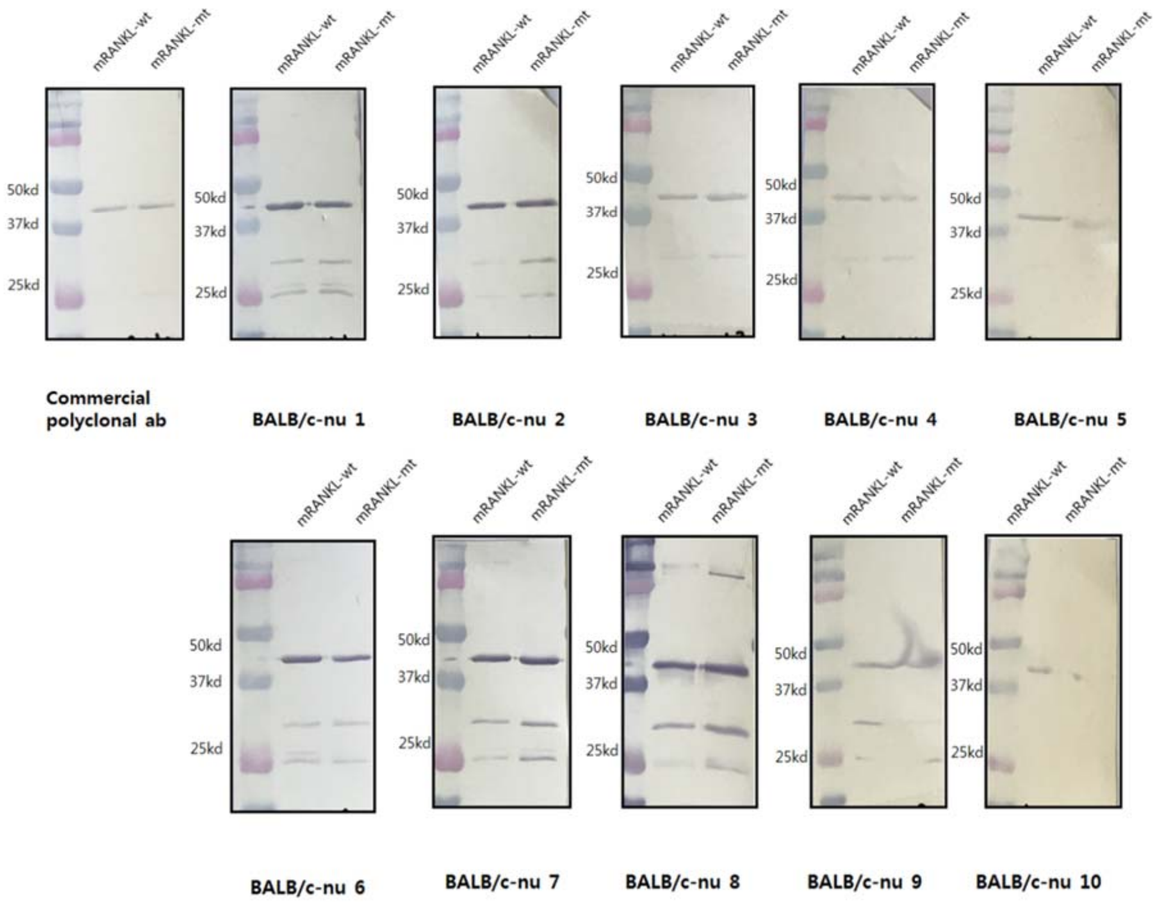

Supple 4. Immunoblot of mRANKL-WT and mRANKL-MT with mtRANKL-immunized BALB-c/nu mouse serum (right) as primary antibodies. The first panel indicated the mRANKL-WT and mRANKL-MT detected by commercial polyclonal RANKL antibody. In entire 10 mice, ani-RANKL for mRANKL-WT and mRANKL-MT were detected.

Figure legends

Figure 1. EMT and metastatic properties of PC3 cells following hRANKL treatment.

(A) Treatment with hRANKL led to a significant increase in the migratory capacity of PC3 cells, as evident from the decrease in the wound gap distance at 48 h. Magnification, 100×; scale bar, 100 μm. The data in the associated graphs are expressed as the mean ± SD. (B) Transwell invasion assays were performed to compare the invasiveness of hRANKL-treated PC3 cells and untreated cells. Treatment with hRANKL led to a significant reduction in the invasiveness of PC3 cells. Magnification, 100×; scale bar, 100 μm. Data in the associated graphs are expressed as the mean ± SD. (C) Real-time quantitative polymerase chain reaction for the analysis of EMT and metastasis markers in hRANKL-treated PC3 cells. β-Actin was used as a loading control. Significant differences were observed at $*p < 0.05$, compared with the control. (D) Expression of EMT and metastasis-related proteins E-cadherin, Vimentin, β-catenin, MMP-9, IL-6, c-MYC, TCF-4 and β-actin in hRANKL-treated PC3 cells was measured by western blotting. β-actin was used as a loading control. (E) Western blot analysis of MAPK phosphorylation in PC3 cells incubated with 1 μg/mL hRANKL for 0, 5, 15 and 30 min. (F) Co-immunoprecipitation of β-catenin and TCF-4 in hRANKL-treated PC3 cells. Results are representative of three separate experiments with comparable results. (G) TOP/FOP luciferase reporter assays in $PC3^{Wild}$ and $PC3^{RANKL+}$ cells. Significant differences were observed at $*p < 0.05$, compared with the control.

Figure 2. Modulation of the EMT and metastatic properties of PC3 cells following overexpression of RANKL.

(A) A cell migration assay was performed to compare wound healing in $PC3^{Wild}$ and $PC3^{RANKL+}$ cells. $PC3^{RANKL+}$ cells showed a significant increase in migratory capacity, as evident from the decreased wound gap at 48 h. Magnification, 100×; scale bar, 100 μm. The data in the

associated graphs are expressed as the mean ± SD. (B) Transwell invasion assays were performed to compare the invasiveness of PC3^{Wild} and PC3^{RANKL+} cells. PC3^{RANKL+} cells showed a significant increase in invasiveness. Magnification, 100×; scale bar, 100 μm. Data in the associated graphs are expressed as the mean ± SD. (C) Real-time quantitative polymerase chain reaction for the analysis of EMT and metastasis markers in PC3^{RANKL+} cells. β-Actin was used as a loading control. Significant differences were observed at *$p < 0.05$, compared with the control. (D) Expression of E-cadherin, Vimentin, β-catenin, MMP-9, IL-6 and β-actin in PC3^{Wild} and PC3^{RANKL+} cells, as measured by western blotting. β-actin was used as a loading control. (E) Immunoprecipitation for β-catenin and TCF-4 in PC3^{Wild} and PC3^{RANKL+} cells. Each blot was obtained under the same experimental conditions and the data are representative of three separate experiments with comparable results. (F) TOP/FOP luciferase reporter assays in PC3^{Wild} and PC3^{RANKL+} cells. Significant differences were observed at *$p < 0.05$, compared with the control.

Figure 3. Effects of immunisation with mutant RANKL.

(A) Comparison of the sequences of hRANKL, mRANKL-WT and mRANKL-MT. (B) *In vivo* experimental flow. Male BALB-c/nu mice (6-weeks-old) were immunised with a subcutaneous injection of test agent (20 μg/kg). (C) Tumour growth and metastases were monitored via *in vivo* bioluminescence imaging of the reporter activity induced by PC3^{Wild}, PC3^{RANKL+} and PC3^{RANKL+} + IM treatment. Data per representative mouse are shown. A large hot-spot of bioluminescence was observed *in vivo* following inoculation of mice with PC3^{RANKL+} cells. Multiple localised and distant metastases were observed *in vivo* after the injection of PC3^{RANKL+} cells into the hearts of nude mice. Coloured bars indicate the bioluminescence signal intensity (photon/s/cm^2/steradian). (D) The bioluminescence photon flux that appeared after inoculation of cells into mice is shown graphically. Data from related graphs are displayed as the mean ±

SD. (E) Survival and (F) metastasis rate in mice from the SHAM, $PC3^{Wild}$, $PC3^{RANKL+}$ and $PC3^{RANKL+}$ + IM groups.

Figure 4. Effects of immunisation with mutant RANKL on PC3-the innoculated metastatic model. (A) Three-dimensional micro-CT images revealed the trabecular bone architecture of the volume of interest in SHAM and mRANKL-immunized (IMMUNIZATION) mouse femurs (n = 10 images taken in total, one image for each mouse). (B) Bone mineral density (BMD) and (C) Serum calcium Level are shown. No significant differences were observed between two groups. (N.S.) (D) representative H&E stained and immunostained images of RANKL and IL-6 in the $PC3^{Wild}$, $PC3^{RANKL+}$ and $PC3^{RANKL+}$ + IM groups. The metastatic tumor cells or immuno-positive cells were indicated by black arrows. Magnification: 200×. Scale bar = 100 μm. (E) Concentration of RANKL in mouse serum. The mean ± SD values were obtained by densitometry, as shown in the analysis. Significant differences were observed at $*p < 0.05$ and $**p < 0.01$ *vs.* control. (F) Serum samples from mice were obtained after immunisation. Anti-RANKL values in $PC3^{Wild}$, $PC3^{RANKL+}$ and $PC3^{RANKL+}$ + IM groups. The mean ± SD values were obtained by densitometry, as shown in the analysis. Significant differences were observed at $*p < 0.05$ and $**p < 0.01$ *vs.* the control. (G) Anti-RANKL values and (H) RANKL concentration in the serum of $RANKL^{-}$immunized mice with metastasis (+) or metastasis (-). Bar graphs show the mean ± standard deviation (SD). Significant differences were observed at $*p < 0.05$, metastasis (+) *vs.* no metastasis (-).

Figure 5. EMT and metastatic properties of PC3 cells treated with immune sera.

(A) Cell migration assays were performed to compare the wound healing capacity of immune serum-treated $PC3^{RANKL+}$ cells and untreated cells. Immune serum treatment decreased the migration capacity of PC3 cells, as evident from the increase in the wound healing gap at 48 h.

Magnification, 100×; scale bar, 100 μm. Data from related graphs are displayed as the mean ± SD. (B) Transwell invasion assays were performed to compare the invasiveness of immunised serum-treated $PC3^{RANKL+}$ and untreated cells. Serum-treated $PC3^{RANKL+}$ cells showed a significant decrease in invasiveness. Magnification, 100×; scale bar, 100 μm. Data in the associated graphs are expressed as the mean ± SD. (C) Real-time quantitative polymerase chain reaction for the analysis of EMT and metastasis markers in $PC3^{RANKL+}$ cells. β-Actin was used as a loading control. Significant differences were observed at *$p < 0.05$, compared with the control. (D) Expression of E-cadherin, Vimentin, β-catenin, MMP-9, IL-6, c-MYC, TCF-4 and β-actin in immune serum-treated $PC3^{RANKL+}$ cells, as measured by western blotting. β-actin was used as a loading control. Similar results were obtained in three independent experiments. (E) Western blot analysis of MAPK phosphorylation levels in serum-treated $PC3^{RANKL+}$ cells. Similar results were obtained in three independent experiments. (F) TOP/FOP luciferase reporter assays in immune serum-treated $PC3^{RANKL+}$ cells. Significant differences were observed at *$p < 0.05$ *vs.* the control. (G) Co-immunoprecipitation of β-catenin and TCF-4 from $PC3^{RANKL+}$ and immune serum-treated $PC3^{RANKL+}$ cells. Each blot was obtained under the same experimental conditions.

Figure 6. Schematic diagram illustrating anti-RANKL-mediated inhibition of RANK signaling. Activation of RANK by RANKL activates AKT and ERK, thereby liberating the active form of β-catenin. Generation of anti-RANKL antibodies by mRANKL-MT inactivates RANKL, thereby downregulating AKT/ERK activity and preventing downstream of β-catenin signaling.

Blot Original Image

Figure 1D Original

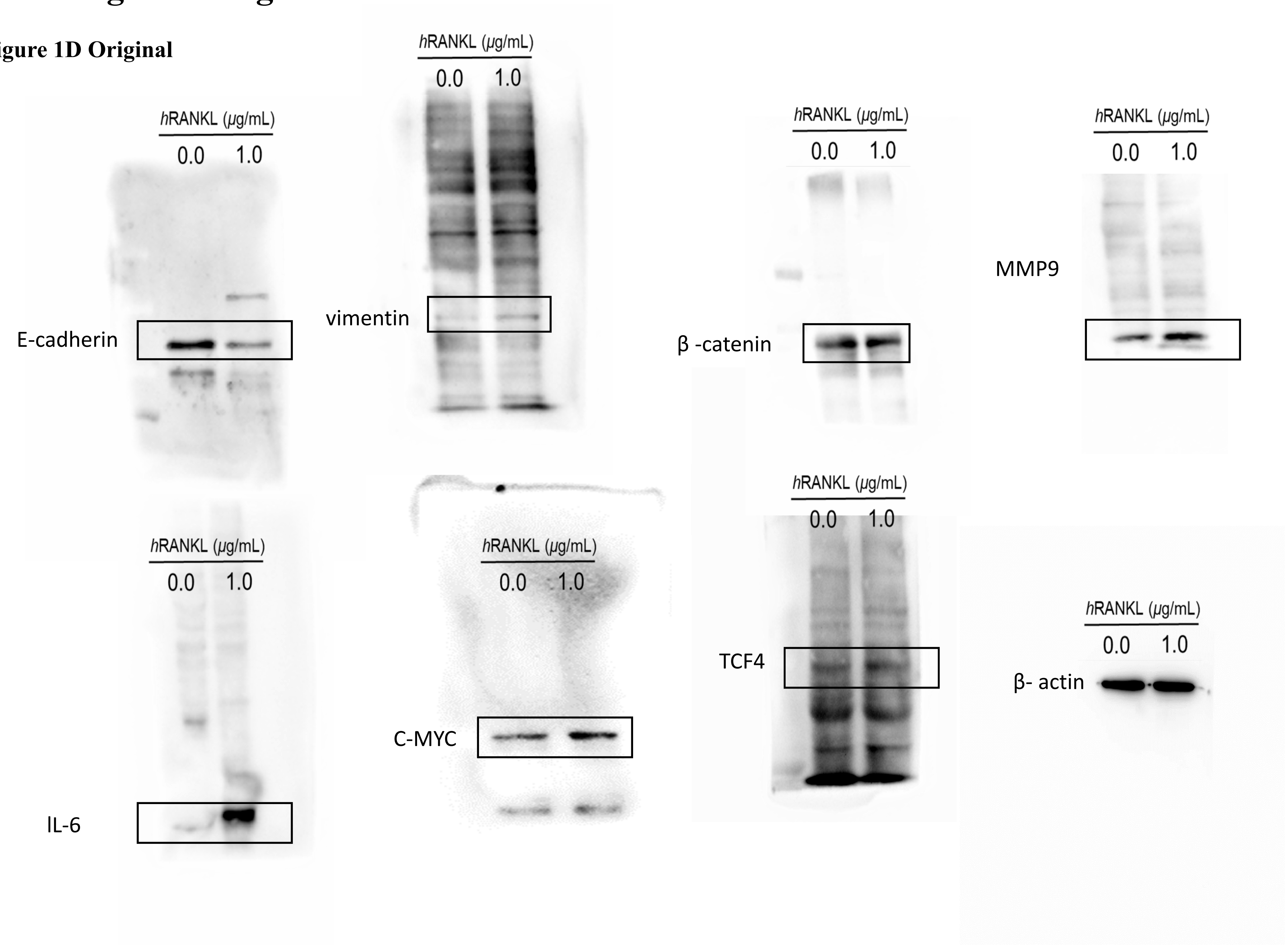

Figure 1E Original

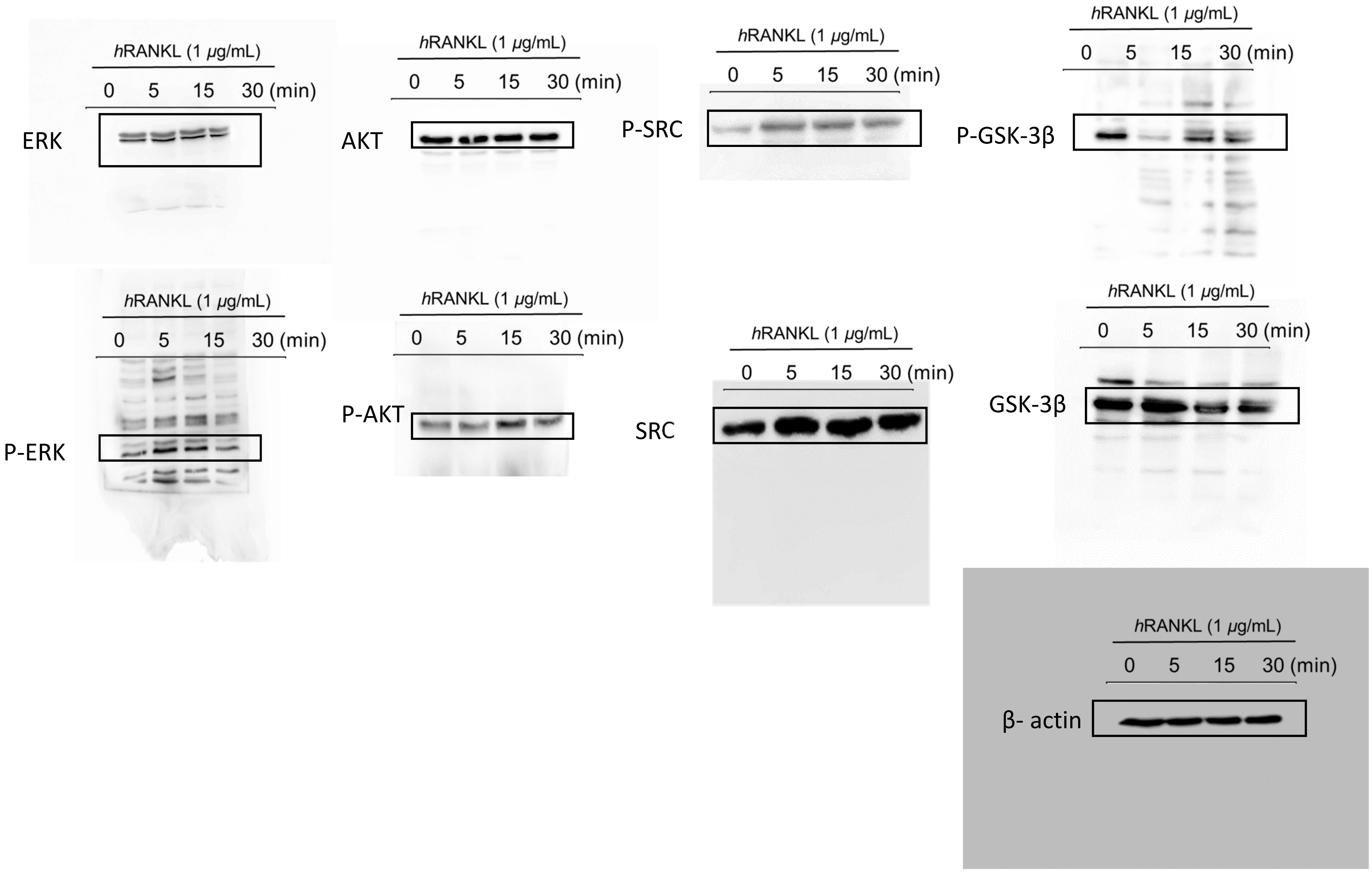

Figure 1F Original

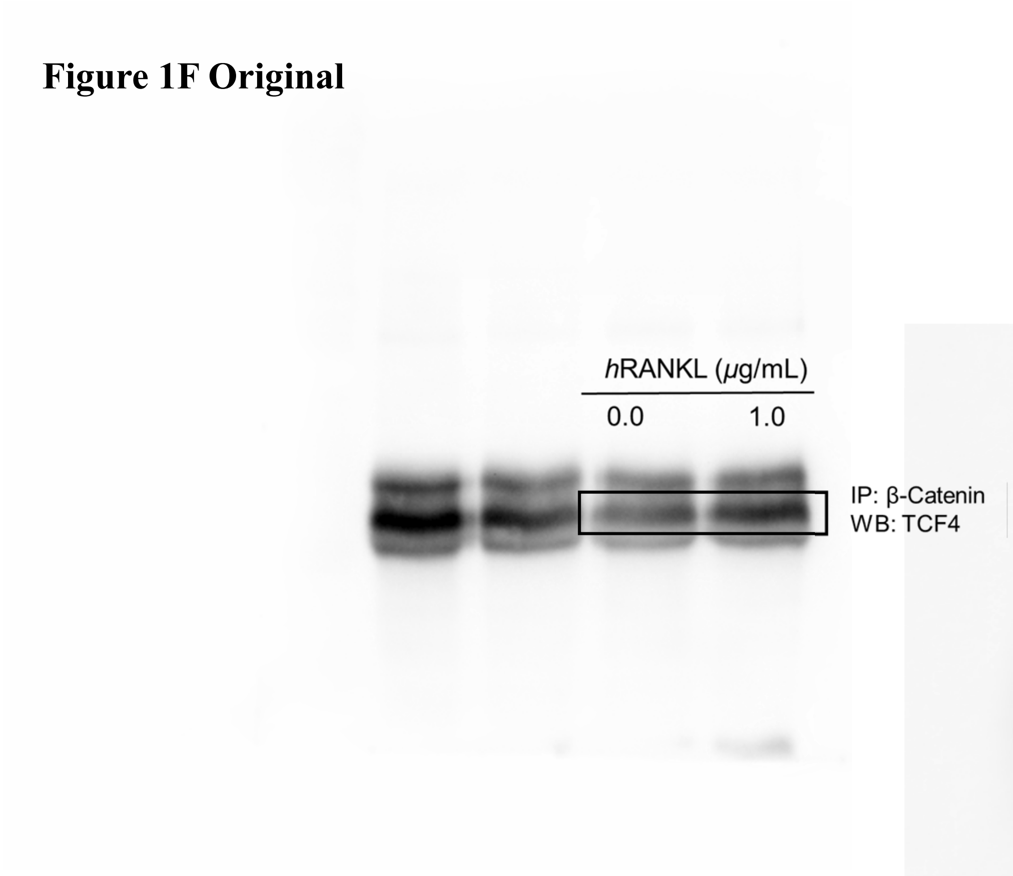

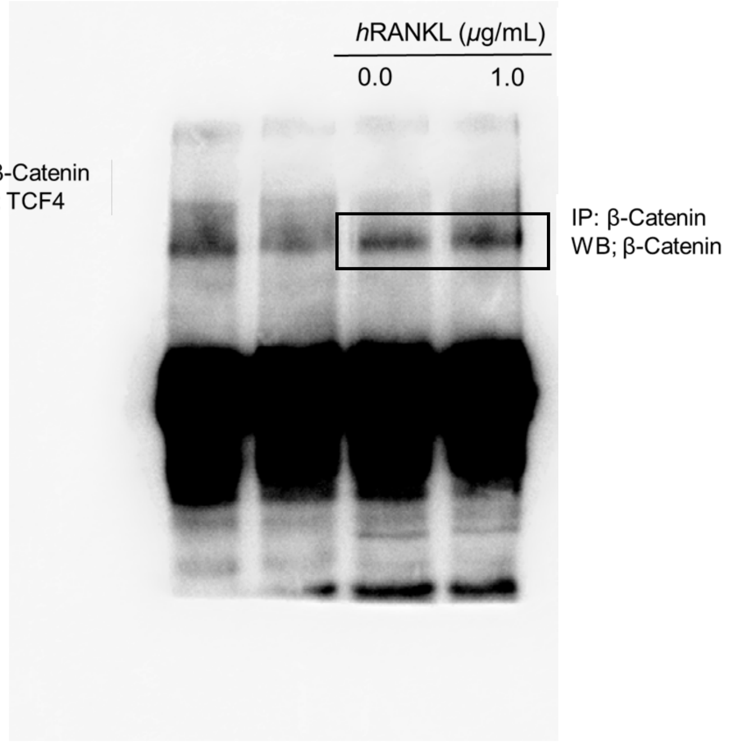

Figure 2D Original

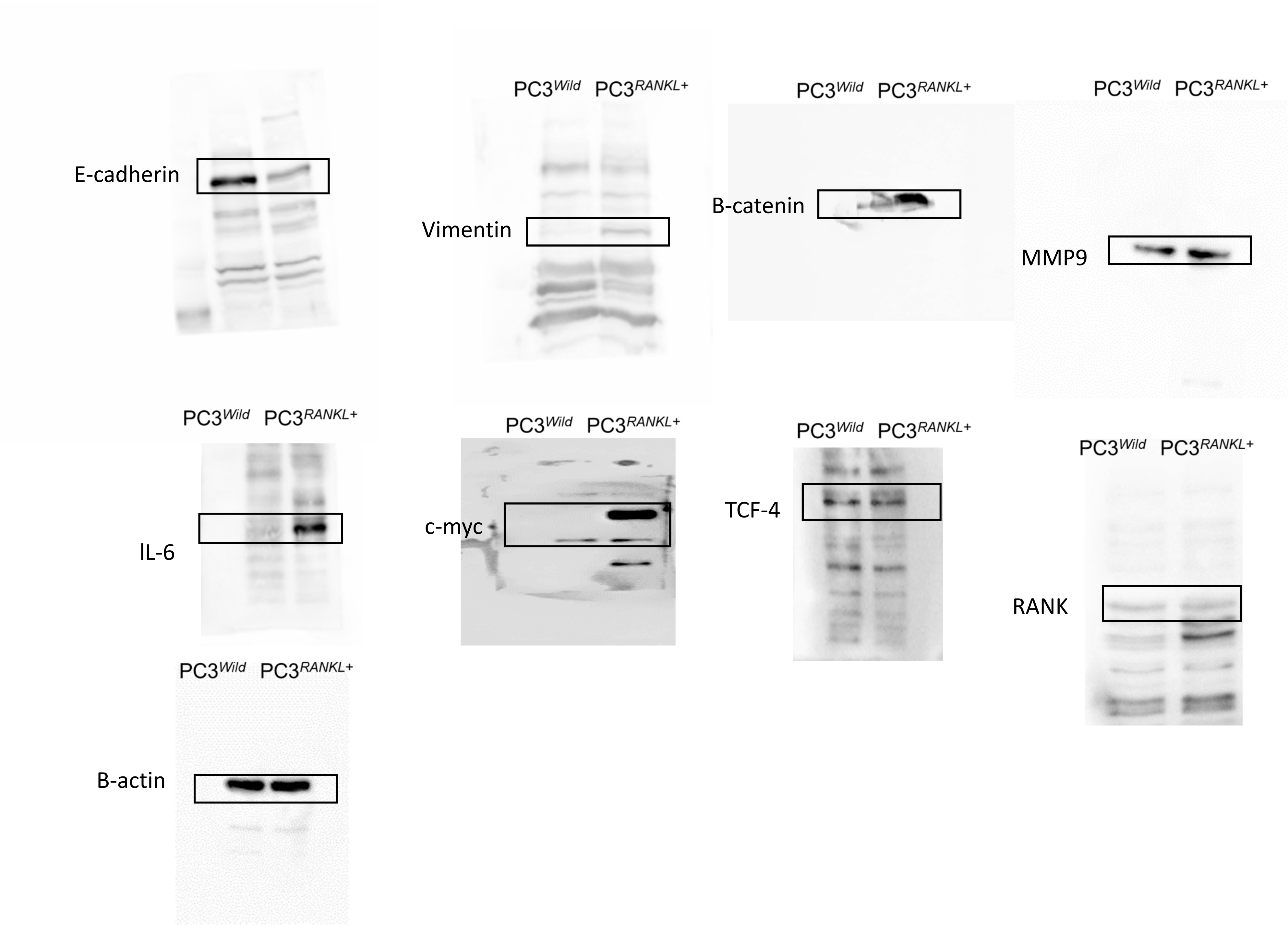

Figure 2F Original

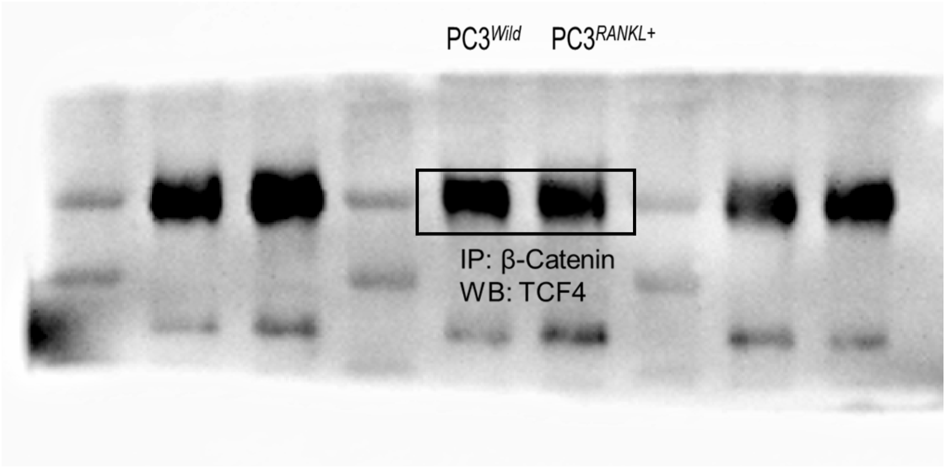

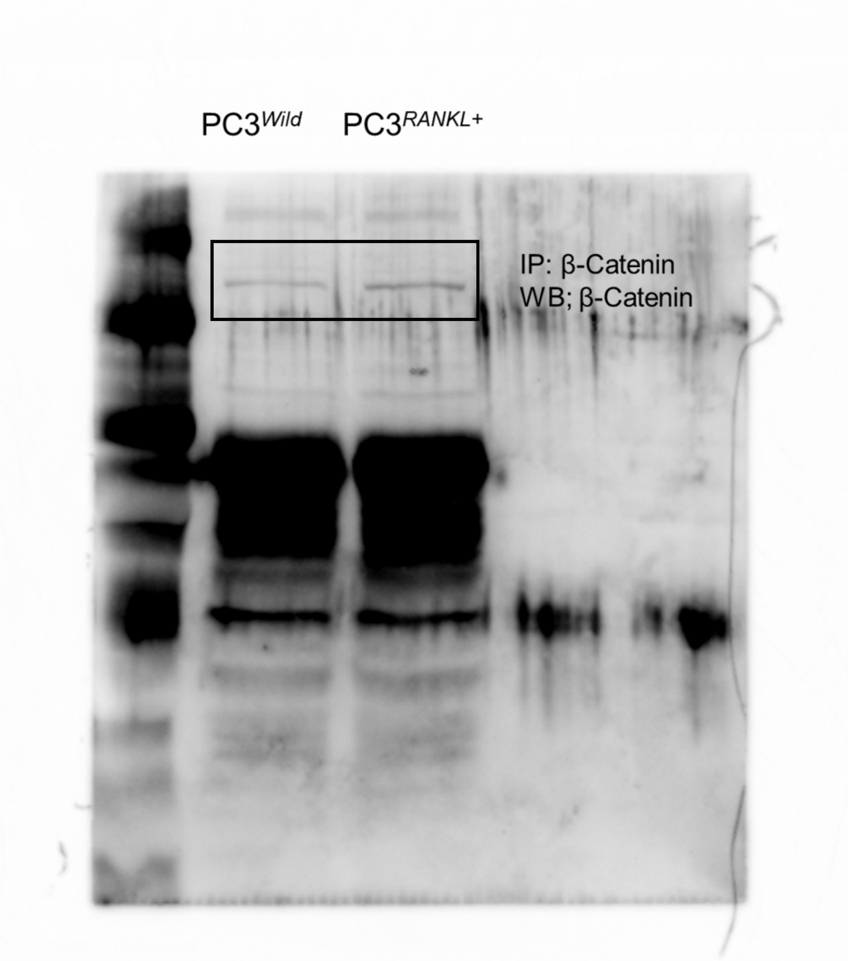

Figure 5D Original

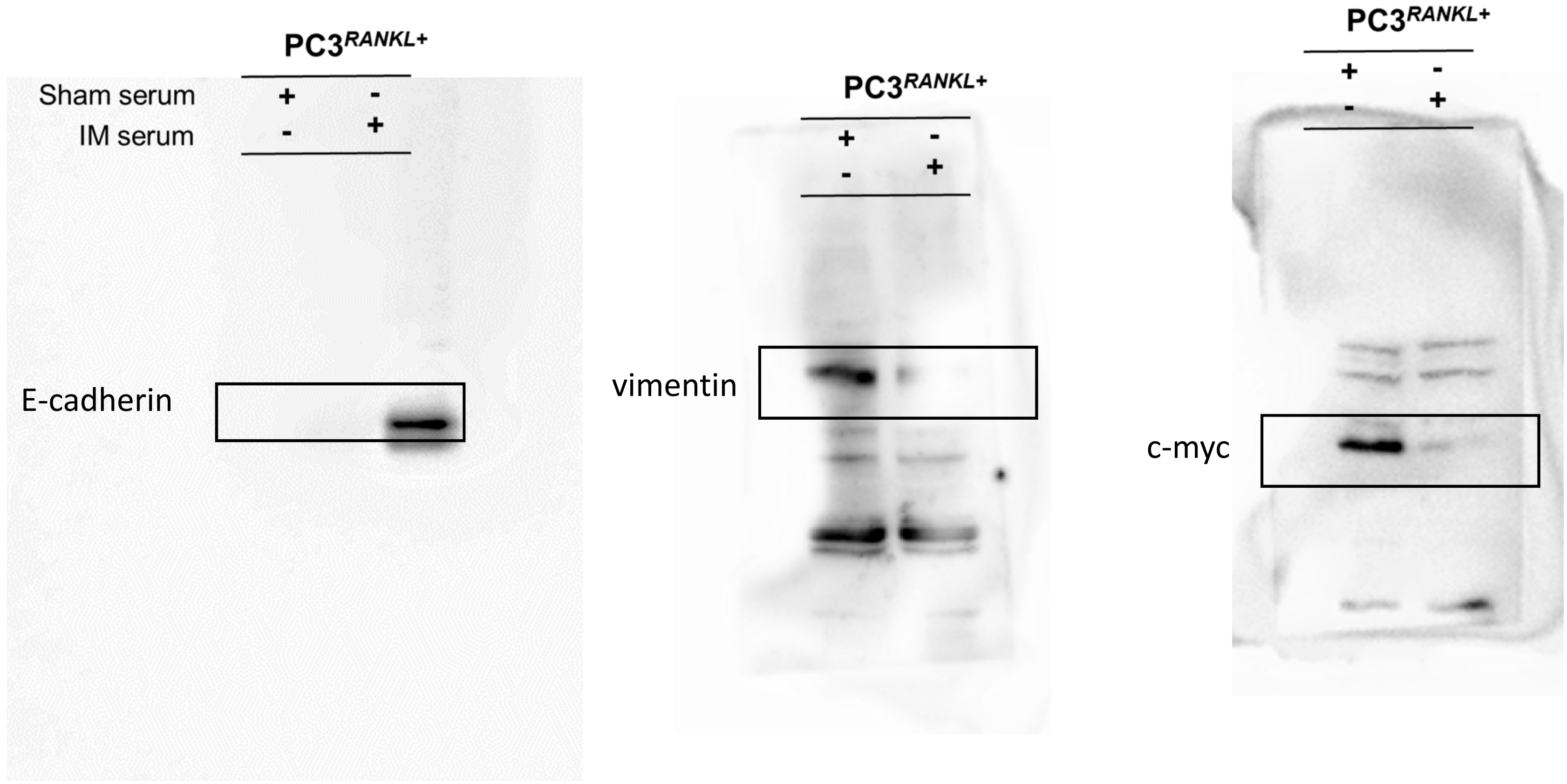

Figure 5D Original

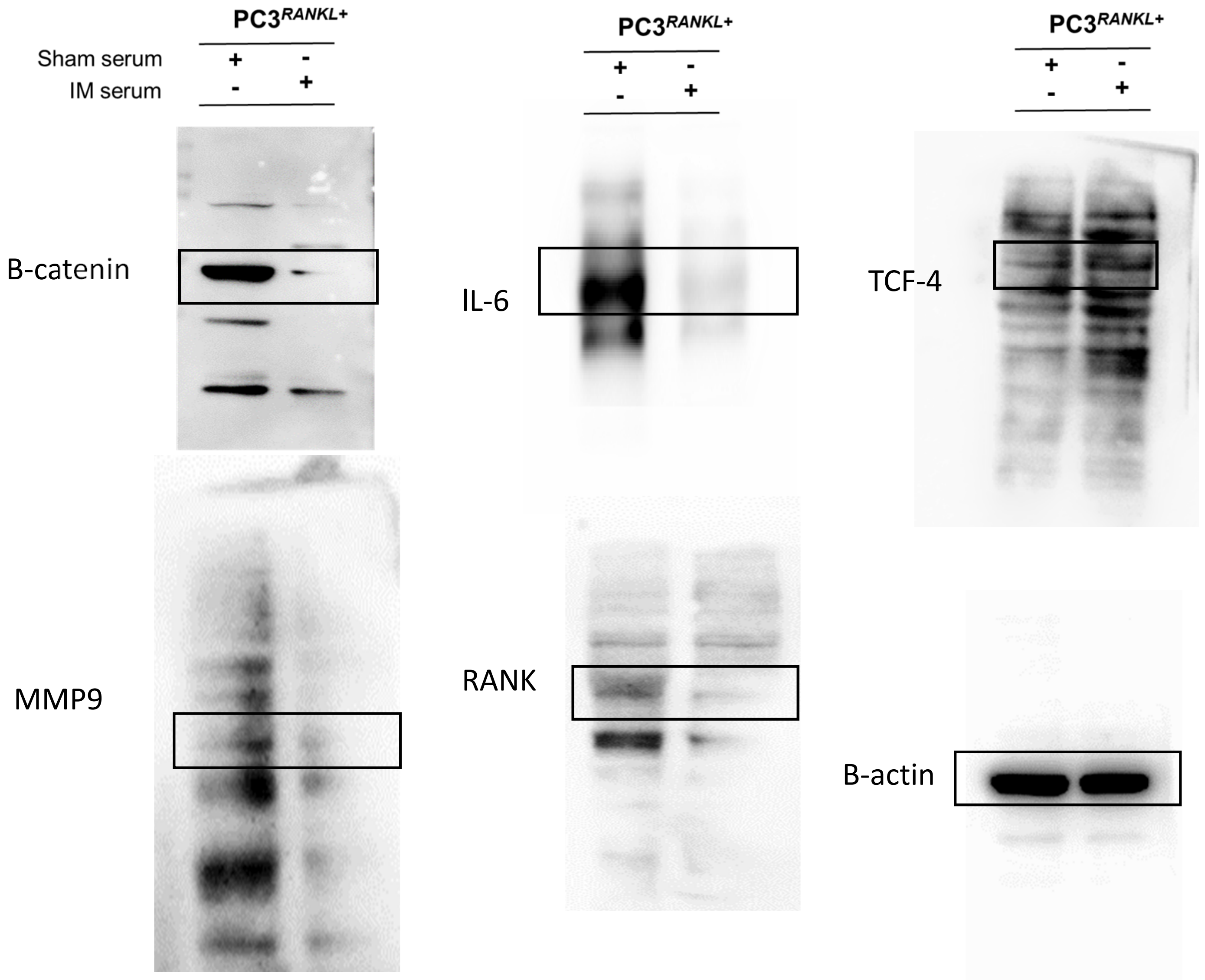

Figure 5E Original

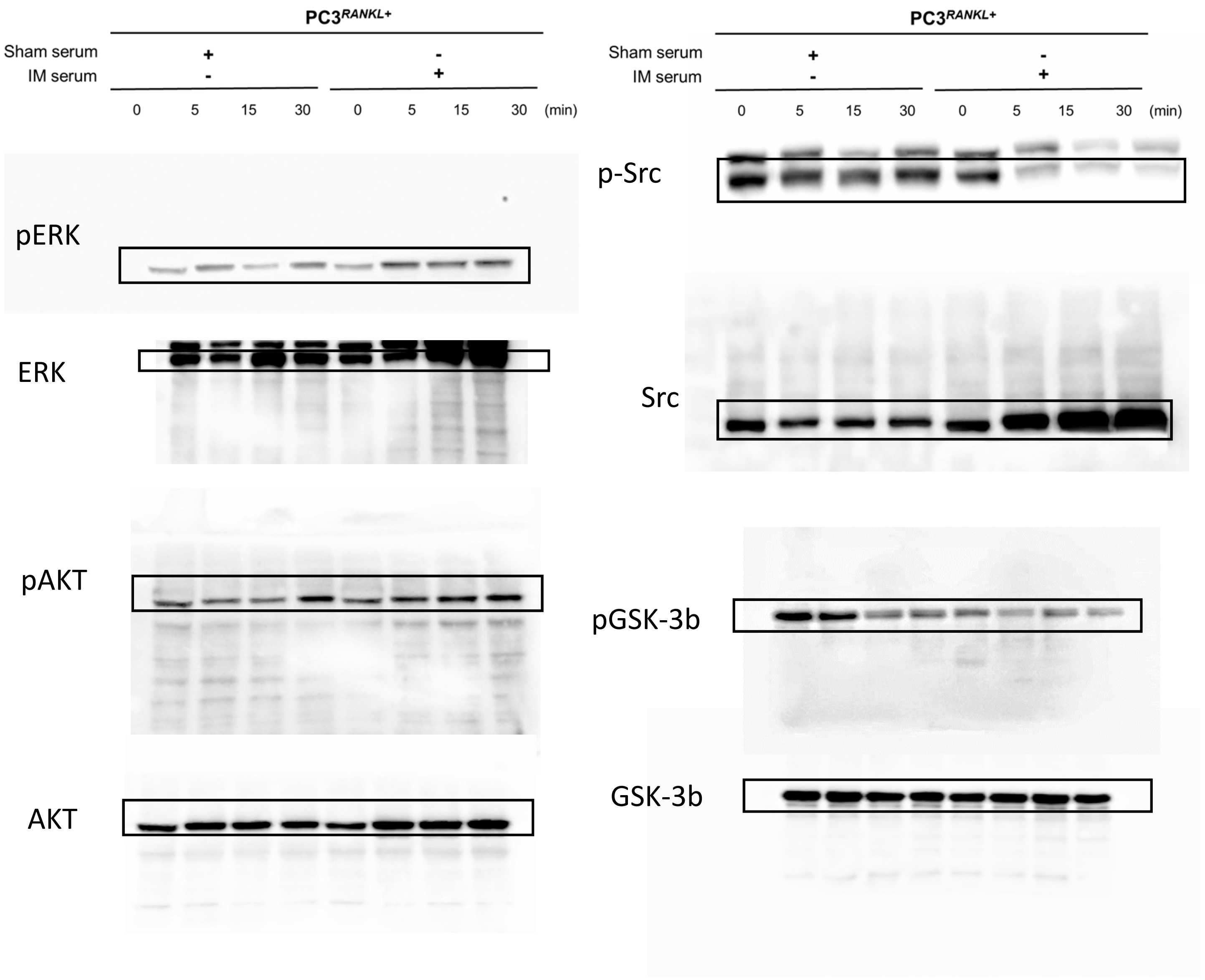

Figure 5E Original

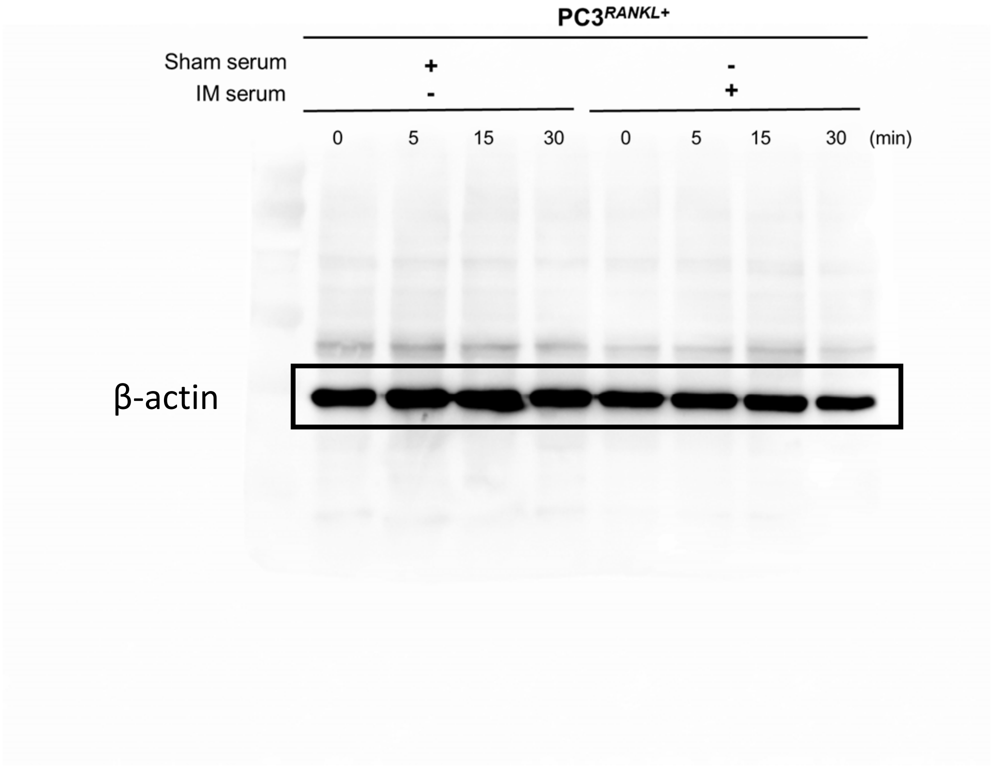

Figure 5G Original

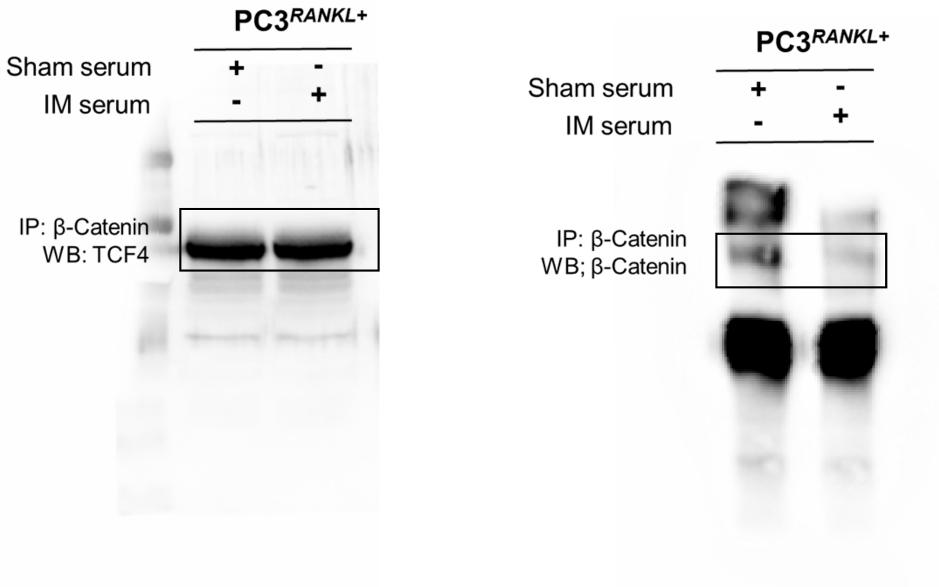

Supple 2 Original

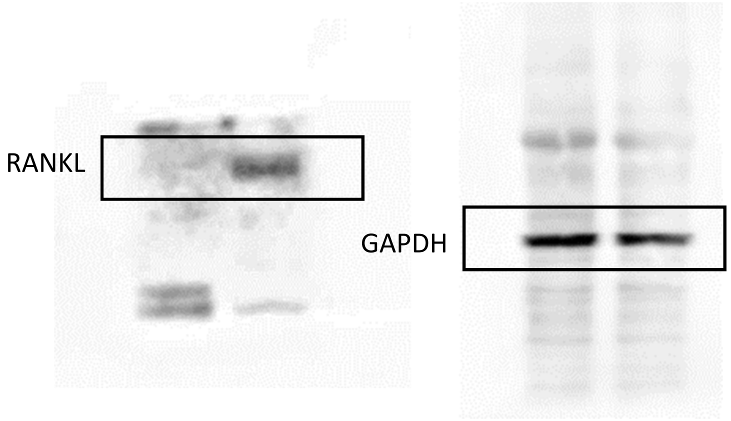

Supplement: Supplementary file 1 — Supplementary Information. [file 41598_2021_91721_MOESM1_ESM.pdf]
